# Lithium Slag as a Supplementary Cementitious Material for Sustainable Concrete: A Review

**DOI:** 10.3390/ma18153641

**Published:** 2025-08-02

**Authors:** Sajad Razzazan, Nuha S. Mashaan, Themelina Paraskeva

**Affiliations:** Mineral Recovery Research Centre (MRRC), School of Engineering, Edith Cowan University, Joondalup, WA 6027, Australia; s.razzazan@ecu.edu.au (S.R.); t.paraskeva@ecu.edu.au (T.P.)

**Keywords:** lithium slag, supplementary cementitious material, sustainable concrete, mechanical properties, durability, waste valorization

## Abstract

The global cement industry remains a significant contributor to carbon dioxide (CO_2_) emissions, prompting substantial research efforts toward sustainable construction materials. Lithium slag (LS), a by-product of lithium extraction, has attracted attention as a supplementary cementitious material (SCM). This review synthesizes experimental findings on LS replacement levels, fresh-state behavior, mechanical performance (compressive, tensile, and flexural strengths), time-dependent deformation (shrinkage and creep), and durability (sulfate, acid, abrasion, and thermal) of LS-modified concretes. Statistical analysis identifies an optimal LS dosage of 20–30% (average 24%) for maximizing compressive strength and long-term durability, with 40% as a practical upper limit for tensile and flexural performance. Fresh-state tests show that workability losses at high LS content can be mitigated via superplasticizers. Drying shrinkage and creep strains decrease in a dose-dependent manner with up to 30% LS. High-volume (40%) LS blends achieve up to an 18% gain in 180-day compressive strength and >30% reduction in permeability metrics. Under elevated temperatures, 20% LS mixes retain up to 50% more residual strength than controls. In advanced systems—autoclaved aerated concrete (AAC), one-part geopolymers, and recycled aggregate composites—LS further enhances both microstructural densification and durability. In particular, LS emerges as a versatile SCM that optimizes mechanical and durability performance, supports material circularity, and reduces the carbon footprint.

## 1. Introduction

### 1.1. Background and Motivation

Concrete remains the most widely used construction material in the world, forming the backbone of modern infrastructure and playing a vital role in supporting economic development. Its versatility, durability, and relatively low cost make it the material of choice for a wide range of applications, from buildings and bridges to roads, dams, and industrial facilities. However, the production of ordinary Portland cement (OPC), the principal binder in concrete, is a major source of carbon dioxide (CO_2_) emissions, responsible for approximately 8% of global anthropogenic emissions [1,2,3,4,5]. This high environmental burden has intensified the search for sustainable alternatives to traditional cementitious materials. Supplementary cementitious materials (SCMs), such as fly ash, ground granulated blast furnace slag (GGBFS), and silica fume, have historically been utilized to partially replace OPC, improving concrete performance while reducing its carbon footprint [1,6,7,8]. A comprehensive inventory of both conventional and emerging low-carbon raw materials has been compiled [9]. Recently, lithium slag (LS), an industrial byproduct generated during lithium extraction from spodumene and lepidolite ores, has attracted significant attention as a promising SCM. The rapid growth of the lithium industry, particularly in Australia and China, has resulted in a substantial increase in LS generation, presenting both an environmental challenge and an opportunity for valorization in cementitious systems [1,2,3,4,5,10]. This aligns with the broad review of mining-waste valorization by Refs. [11,12]. The application of mining waste materials in potential geotechnical applications—such as soil stabilization, backfill reinforcement, and earth-structure construction—has also been investigated by Ref. [13]. Further, the valorization of sludges from aggregate production into construction materials—including filler, supplementary cementitious materials, and lightweight aggregates—has been comprehensively examined by Ref. [14]. Similarly, the incorporation of stabilized waste soil from landfill mining as a partial replacement for fine aggregate in concrete—up to 30% by volume—has been shown to improve compressive, tensile, and flexural strengths as well as durability [15]. Beyond lithium slag, several recent SCM innovations demonstrate the broader potential of industrial by-products in concrete: For example, Ref. [16] performed a macro-micro investigation of stabilized sludge as a subgrade filler using a ternary blend of steel slag, fly ash, and calcium-carbide residue, achieving unconfined compressive strengths above 1.5 MPa at 7 days while significantly reducing embodied CO_2_. The authors of Ref. [17] applied a combined AHP–entropy weighting framework to recycled carbon-fiber-reinforced cement mortar, finding that just 0.8 wt% carbon fiber yielded a 19% higher composite benefit index—driven by 35–45% gains in tensile and flexural performance—without sacrificing environmental metrics. The authors of Ref. [18] conducted in situ dynamic response tests on highway transition sections stabilized with foamed concrete, reporting exponential attenuation of traffic-induced stresses and proposing new stiffness-based smoothness criteria for sustainable pavement design. Furthermore, Ref. [19] investigated the modulation of initial CaO/Al_2_O_3_ and SiO_2_/Al_2_O_3_ ratios in slag/fly ash-based geopolymers for stabilized clay, demonstrating synergistic effects that significantly enhanced early-age compressive strength and durability while further reducing the CO_2_ footprint.

### 1.2. Scope and Significance of Lithium Slag (LS) as an SCM

Lithium slag is primarily composed of silica (SiO_2_) and alumina (Al_2_O_3_), with varying contents of calcium oxide (CaO), lithium oxide (Li_2_O), and minor phases, typically exhibiting a partially amorphous structure that imparts pozzolanic activity [1,2,4,10,20,21]. Similar applications of metallurgical wastes in concrete have been systematically reviewed by [22], and gold tailings sludge has also demonstrated cement-replacement potential [23].

Several studies have demonstrated that incorporating LS enhances the mechanical performance of concrete, notably increasing compressive strength, tensile strength, and flexural strength [4,6,7,21,24,25,26,27,28,29,30,31,32,33]. These improvements are attributed to enhanced hydration reactions, secondary C-S-H and C-A-S-H gel formation, and densification of the interfacial transition zone (ITZ). Durability improvements associated with LS use have also been widely reported, including reductions in chloride ion penetration, sulfate attack, drying shrinkage, and freeze–thaw damage [4,6,7,20,24,25,26,28,31,33,34,35,36]. The finer particle size and pozzolanic reaction contribute to a refined pore structure and improved impermeability [37].

Several investigations have focused on the use of LS in alkali-activated and geopolymer systems, either alone or in combination with other SCMs such as fly ash, metakaolin, and slag [4,5,8,10,20,26,38]. Applications in alkali-activated and 3D-printed systems have been preliminarily assessed [39]. These studies have shown that LS enhances the polymerization reaction, improves setting times, and increases mechanical strength and chemical stability. A broader review of 3D-printed concrete with industrial by-products is provided by [40]. Synergistic effects of combining LS with traditional SCMs in blended systems have also been reported, contributing to enhanced workability, durability, and cost-efficiency [4,6,10,20,38,41,42]. Moreover, some studies have specifically explored LS’s role in improving the performance of recycled aggregate concrete [10,26,43].

Microstructural analyses have also been extensively conducted to understand LS’s role at the microscopic level. Several researchers have studied hydration product formation, pore structure refinement, ITZ densification, and crystalline/amorphous phase development in LS-modified systems [1,2,20,21,27,28,33,44]. These studies confirm that LS incorporation leads to a denser matrix, higher volumes of C-S-H and C-A-S-H gels, and a refined pore size distribution, all of which contribute to improved mechanical and durability performance. Microstructural analyses via SEM and XRD confirm the formation of secondary C–S–H phases, paralleling findings in other tailings-based materials [45].

The environmental and sustainability aspects associated with LS utilization have been qualitatively discussed across many studies. Valorizing LS as a cement replacement material reduces landfill burdens, conserves natural raw materials, and potentially lowers the embodied energy of concrete production [1,2,4,5,6,10,20,38,41,46]. However, most studies emphasize that comprehensive quantitative assessments, such as life-cycle assessment (LCA), remain lacking and should be a focus of future research [4,41].

Despite substantial progress in demonstrating the benefits of lithium slag (LS) in concrete, its reliable, large-scale implementation remains hindered by several critical challenges. Our comprehensive review reveals pronounced source-dependent variability in LS chemistry that translates into unpredictable pozzolanic reactivity, fresh-state workability, and strength development. Moreover, our analysis of various replacement levels highlights the importance of a balanced mix design: insufficient LS yields marginal gains, while excessive substitution can compromise performance. Compounding these technical hurdles are significant research gaps, including the lack of rigorous life-cycle assessments, limited evaluation of LS in specialized contexts (such as fatigue resistance or high-temperature applications), and absence of standardized processing and quality-control protocols. By systematically correlating compositional parameters with key performance indicators and outlining evidence-based mix-design frameworks, this review establishes clear guidelines to interpret and synthesize previously reported characterizations of lithium slag (LS) and to support its effective deployment in sustainable concrete systems—paving the way for its confident, industry-scale adoption.

## 2. Review Methodology

To ensure a systematic and transparent review process, a structured methodology was implemented for identifying and analyzing relevant studies on lithium slag (LS) as a supplementary cementitious material in concrete applications.

### 2.1. Databases and Search Strategy

The literature search was conducted through Scopus, Web of Science, ScienceDirect, and Google Scholar databases. Searches were performed using combinations of keywords, including “lithium slag,” “supplementary cementitious material,” “SCM,” “concrete,” “durability,” “mechanical properties,” “microstructure,” and related terms.

### 2.2. Inclusion and Exclusion Criteria

The studies included in this review were published between 2011 and 2024, written in English, and consisted of peer-reviewed journal articles, conference proceedings, or technical reports. Additionally, the included studies explicitly focused on the use of lithium slag (LS) in cementitious materials and provided quantitative data on fresh-state properties, mechanical properties, durability, or microstructural characteristics. In contrast, non-English publications, review papers, theses, dissertations, opinion-based articles, and papers lacking quantitative experimental data or not directly addressing LS in concrete were excluded.

### 2.3. Screening and Selection Process

Initially, around 100 papers were identified. After removing duplicates and screening titles and abstracts for relevance, approximately 70 papers remained. A detailed full-text evaluation narrowed this number to around 55. Finally, 42 papers fully meeting the defined criteria were included for detailed analysis.

### 2.4. Data Extraction and Synthesis

Key information extracted included LS chemical composition, dosage, concrete types, testing methods, and experimental results. Due to variations in LS sources and methodologies across studies, descriptive statistics, comparative analyses, and graphical summaries were used to synthesize and present the findings.

### 2.5. Limitations and Boundary Conditions

The review was limited to English-language literature, which may have excluded relevant regional studies. Additionally, the variability in LS chemical composition from different geographical sources should be considered when interpreting generalized conclusions.

## 3. Analysis of the Literature Review

### 3.1. Research Trends and Publication Growth

A significant challenge arises from the fact that all the reviewed studies are based on LS obtained from various industrial sources, with each originating from different mines located in different parts of the world. As a result, the chemical composition of LS is not unique or consistent, leading to variability in its behavior when used in concrete. This inconsistency in chemical composition means that LS obtained from one region may behave differently from LS sourced from another, making it difficult to generalize research findings across all LS-based concrete applications. Consequently, the results of these studies may not reliably translate to industrial use of LS-based concrete made from local lithium mines. The variability in LS composition can pose challenges for industry professionals, as they may struggle to predict how locally sourced LS will interact with concrete, leading to uncertainty in performance outcomes. Therefore, while LS holds potential, careful consideration of its source and composition is necessary for practical applications, and additional localized testing may be required to ensure reliable performance.

Another critical aspect that has received limited attention in the literature is the applicability of LS-based concrete in specialized concrete applications, such as its uses in the pavement industry. However, the effect of LS on the specific mechanical and durability properties such as fatigue resistance, freeze-thaw resistance, and thermal expansion coefficient required for rigid pavement concrete has not been extensively studied.

**Figure 1:**
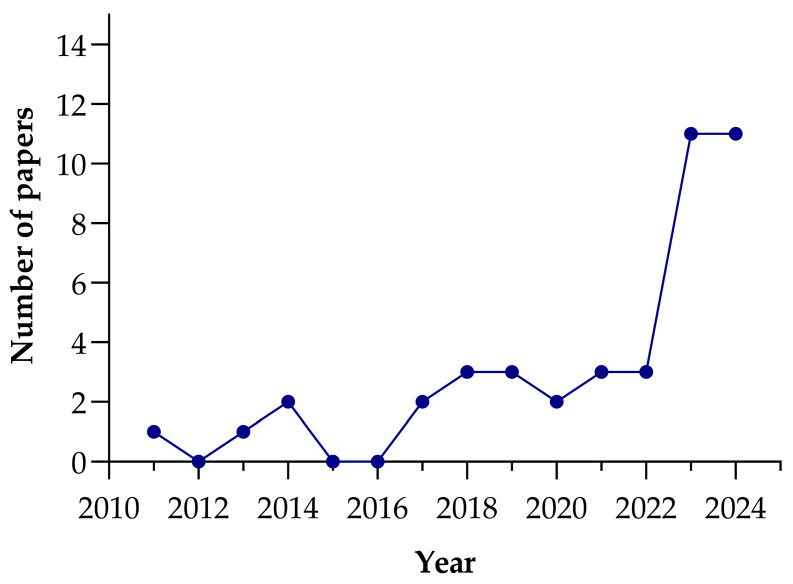
Number of studies on LS concretes in different years.

According to Figure 1, starting from 2011, when only one study was published, the research gradually gained momentum, with slight increases in 2014 and 2017. For instance, in 2011, there was only one study, and in the following years, we noticed some gaps, such as in 2012, 2015, and 2016, where the authors could not find any published studies specifically related to LS in concrete application. However, this does not necessarily mean that there were no studies at all; as far as the authors know, no significant research focusing on lithium slag in concrete applications was conducted or published during those years. However, from 2018 onwards, there has been a steady rise in the number of publications, reflecting the growing interest and potential in using lithium slag as a supplementary cementitious material.

In particular, the years 2023 and 2024 show a remarkable surge, with 11 and 10 studies, respectively. This sharp increase demonstrates that lithium slag is gaining recognition as an important area of investigation in sustainable construction and material innovation. This trend aligns with the increasing focus on environmental sustainability and the search for eco-friendly alternatives in the construction industry.

### 3.2. Chemical Composition of Lithium Slag in Previous Studies

**Figure 2:**
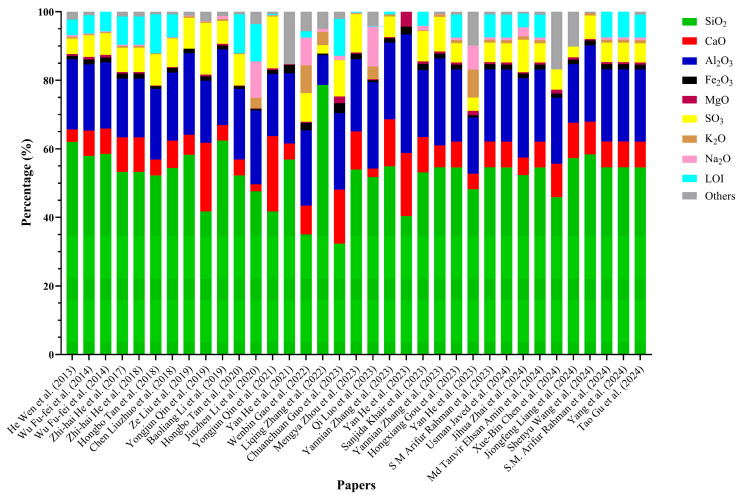
Chemical composition of lithium slag based on various papers [2,6,8,10,20,21,24,25,26,27,28,29,30,31,32,34,38,41,44,47,48,49,50,51,52,53,54,55,56,57,58,59,60,61,62].

As evidenced in Figure 2, the chemical composition of lithium slag exhibits significant variability across different studies. This variation in key components, such as SiO_2_, CaO, and Al_2_O_3_, underscores the inconsistencies inherent in lithium slag derived from diverse sources or production processes. For instance, SiO_2_ concentrations range from as low as 32% to as high as 78%, while CaO content fluctuates between 2% and 22%. The impact of variations in the chemical composition of LS on concrete properties remains unclear due to the absence of a systematic investigation.

These discrepancies pose a challenge when evaluating lithium slag as a sustainable material for incorporation into concrete mixes. The variation in chemical composition has direct implications for the mechanical properties and durability of the resultant concrete, necessitating further investigation into the material’s consistency and performance in concrete applications.

**Figure 3:**
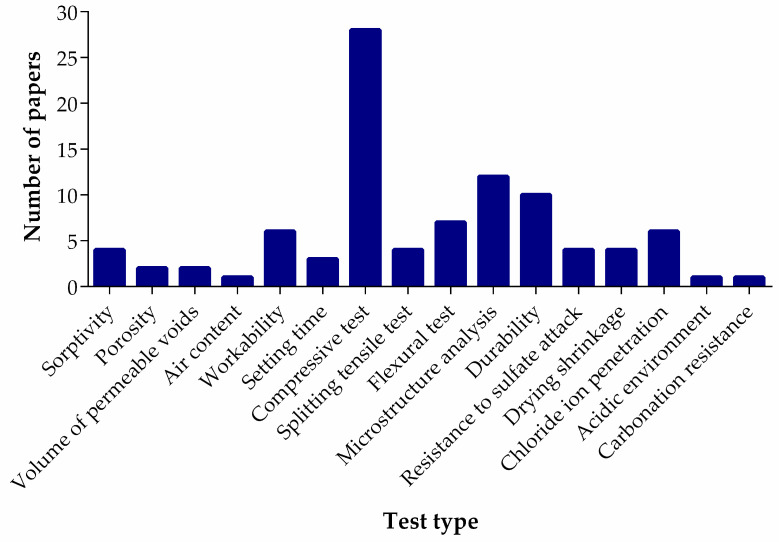
Number of tests conducted on LS concrete.

Figure 3 represents key data from various studies on lithium slag (LS) concrete, highlighting critical tests conducted by other researchers. The data includes results from four sorptivity tests, which measure the water absorption capacity of LS concrete, as well as two porosity tests and two tests on the volume of permeable voids, providing insight into the movement of fluids within the concrete. Air content was assessed in one test, while six tests examined workability, and three tests focused on the setting time of the concrete.

Regarding the mechanical properties, 28 compressive strength tests were conducted, alongside 4 splitting tensile tests and 7 flexural tests. Furthermore, 12 microstructural tests provided valuable information on the internal structure of the concrete. Durability was evaluated through 10 tests, with 4 tests specifically addressing sulfate attack resistance. Additionally, four drying shrinkage tests and six tests of chloride ion penetration were analyzed. Finally, one test each was conducted to assess performance in acidic environments and carbonation resistance.

Although this review highlights the wide variability in LS chemical composition—particularly in the content of SiO_2_, Al_2_O_3_, and CaO—no quantitative model was developed to correlate these variations with pozzolanic activity or performance outcomes. This limitation is primarily due to inconsistencies in data reporting across studies. Many papers provide only partial oxide composition, lack standardized pozzolanic reactivity tests, or omit complementary performance metrics, such as compressive strength or permeability, under the same mix design. Moreover, differences in characterization techniques (e.g., XRF vs. EDS) and testing protocols further complicate comparative analysis. As a result, while trends can be qualitatively observed, a robust multivariate analysis could not be performed within the scope of this review. Future studies should aim to report complete and consistent datasets to enable statistically meaningful correlations between LS composition and its reactivity or performance in concrete.

### 3.3. Experimental Investigations of LS Concrete in Previous Studies

**Figure 4:**
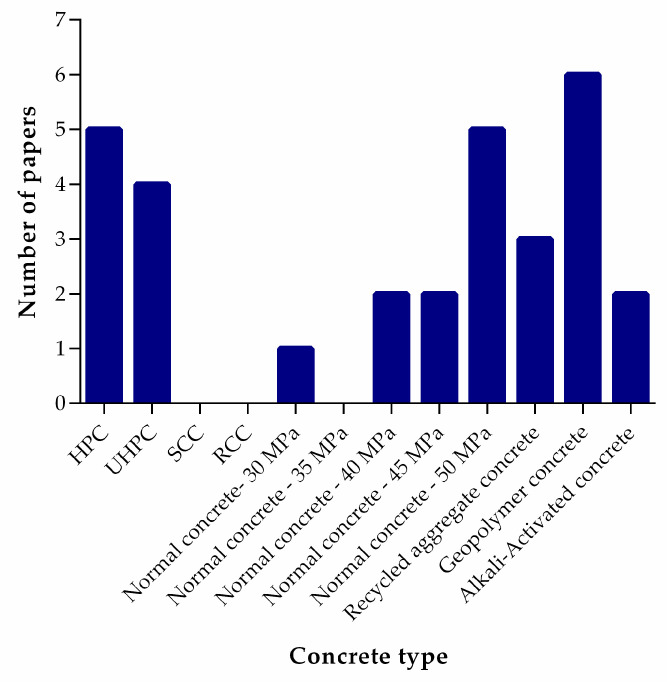
Number of studies on different concrete types (from 2011 to 2024).

Figure 4 clearly demonstrates that most investigations to date have centered on conventional (normal) concrete, reflecting both the ubiquity of this material in construction practice and the availability of standardized testing methods (e.g., ASTM and EN protocols) that facilitate direct comparison of workability, strength development, and durability improvements imparted by lithium slag. By establishing a robust baseline in normal concrete, researchers have been able to quantify key performance metrics—such as compressive strength gain, pore structure refinement, and resistance to chloride ingress—under well-controlled conditions, thus laying the groundwork for broader application of LS as a supplementary cementitious material.

Following normal concrete, the next most extensively studied systems are ultra-high-performance concrete (UHPC) and geopolymer concrete. In UHPC formulations, the incorporation of LS fine particles and reactive silica-alumina phases have been shown to contribute to the material’s hallmark ultra-dense microstructure, leading to exceptional mechanical properties and early-age strength gains [2]. Geopolymer concrete research, on the other hand, leverages LS’s aluminosilicate-rich composition to participate in alkali-activation processes, offering a low-carbon alternative to both Portland cement and conventional SCMs like fly ash and slag [38]. These studies have elucidated how LS affects setting times, polymerization kinetics, and long-term chemical stability in alkali-activated binders, thus expanding the potential palette of low-carbon binders available to the construction industry.

By contrast, applications of LS in more specialized formats, namely roller-compacted concrete (RCC) and self-compacting concrete (SCC), remain underexplored, as evidenced by the relatively small number of studies in these categories. Given RCC’s growing prominence in pavement construction and the critical role of compaction characteristics and fatigue resistance under cyclic loading, the systematic evaluation of LS’s influence on mixture cohesiveness, stiffness development, and long-term performance under traffic loading represents a promising research direction. Similarly, SCC’s reliance on precise rheological control to achieve high flowability without segregation underscores the need to investigate how varying LS content alters viscosity, yield stress, and stability in self-leveling applications. Beyond these areas, emerging niches such as 3D-printed concrete and fiber-reinforced systems have also received scant attention; extending LS research into these domains can uncover new opportunities for tailoring printability, build-up robustness, and interfacial bonding in advanced construction technologies.

## 4. Reviewing the Outcomes and Findings

### 4.1. LS Replacement Levels in Various Concrete Types

**Figure 5:**
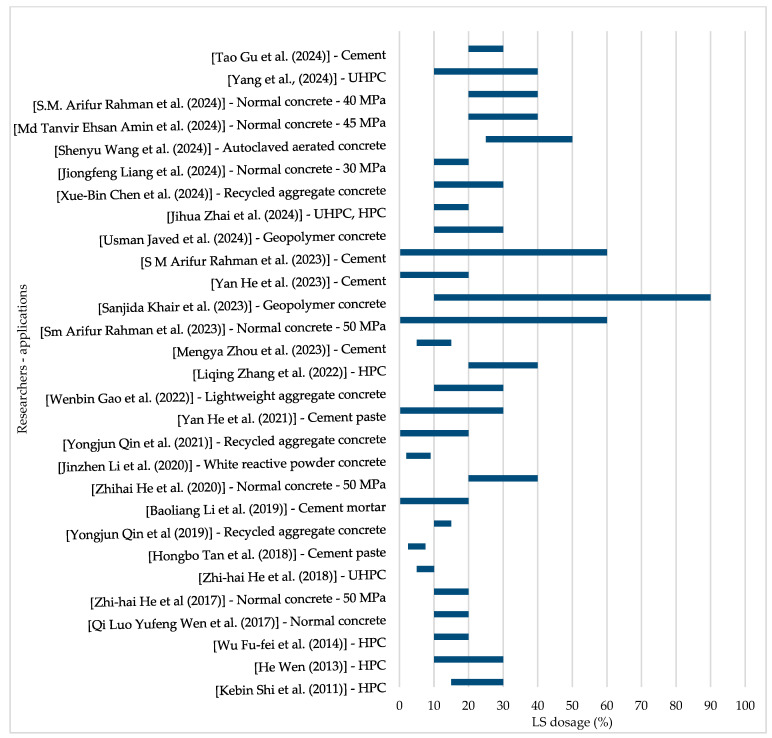
Variation in LS levels used by other researchers with the type of concrete they studied [1,2,6,7,8,10,20,21,24,26,27,28,29,30,31,32,34,38,41,44,47,48,49,50,51,52,53,54,55,56,57,58,59,60,61,62,63,64,65].

Figure 5 illustrates the variation in lithium slag (LS) dosage levels adopted by different researchers, highlighting a broad range from minimal replacements to values exceeding 90%. This variation underscores the diverse experimental approaches used to evaluate LS as a supplementary cementitious material, reflecting differences in research objectives, material properties, and performance criteria. Understanding these variations is crucial for selecting an optimal LS dosage in this study, ensuring a balance between mechanical performance, durability, and sustainability. The findings from this review will help establish a practical LS replacement range for concrete and rigid pavement applications.

**Figure 6:**
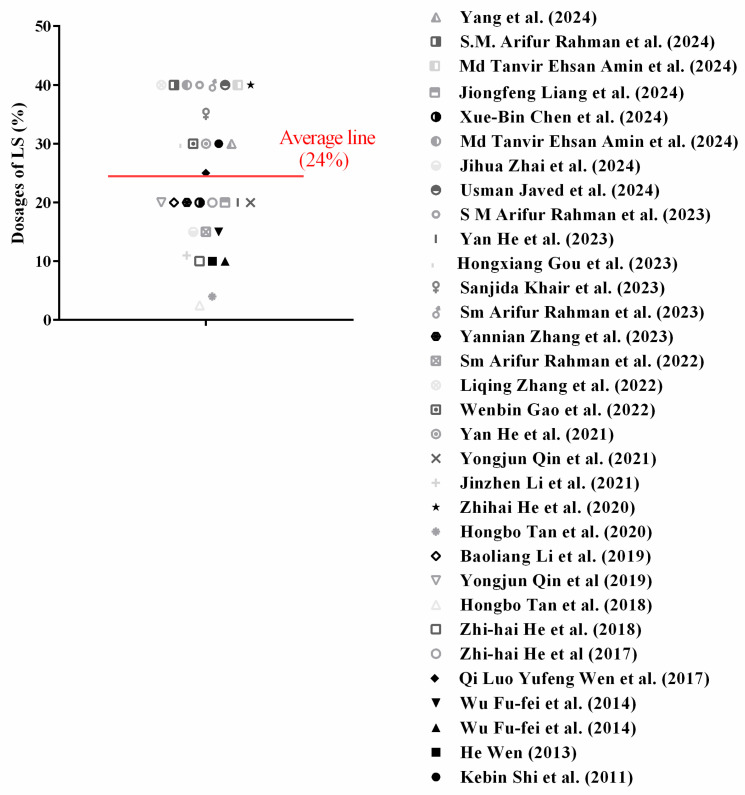
Optimum dosage of LS reported by various papers [1,2,6,7,10,21,24,25,27,28,29,31,32,34,38,41,44,47,48,49,50,51,52,57,58,60,61,62,63,64,65].

As shown in Figure 6, the reported LS dosages vary significantly, with an average value of 24%. This suggests that while higher dosages have been explored, a balanced approach should consider performance optimization in terms of mechanical properties, durability, and sustainability. The findings from this review will guide the selection of appropriate LS replacement levels in this research, ensuring practical and efficient utilization of LS in cementitious materials.

**Figure 7:**
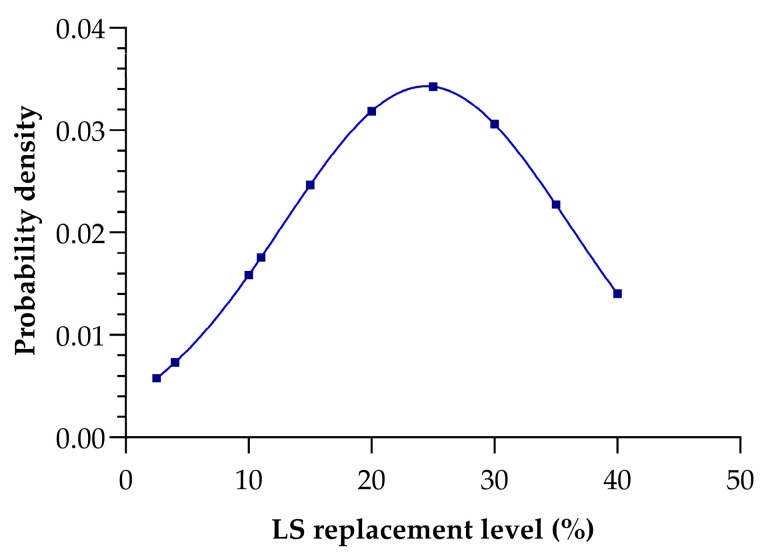
Normal distribution of LS replacement percentage.

Figure 7 presents the normal distribution of lithium slag (LS) replacement percentage, illustrating the probability density of different dosage levels in the studied mix designs. The distribution exhibits a peak of around 25–30% LS replacement, suggesting that this range is the most frequently used or optimal level within the dataset. The average LS replacement percentage is 24%, with a standard deviation of 11%, indicating a moderate spread of data around the mean. According to the properties of a normal distribution, approximately 68% of the data falls within one standard deviation of the mean (13% to 35% LS replacement) and approximately 95% of the data falls within two standard deviations of the mean (2% to 46% LS replacement). The probability density is lower at both lower and higher replacement levels, suggesting that extreme values (below 10% and above 35%) are less common or less effective in the context of the study. The smooth curve reflects the overall trend, highlighting the central tendency and variability of the LS dosage percentages used in the research.

### 4.2. Fresh Properties of LS-Based Concrete

The study conducted by the authors of Ref. [27] provided a detailed evaluation of the fresh properties of concretes incorporating lithium slag (LS) as a partial cement replacement at 20%, 40%, and 60% levels. The key parameters assessed included air content, compaction factor, water penetration capacity (water retention capacity), fresh density, and setting time, which are essential indicators of workability, placement performance, and early-age hydration behavior. Air Content: The air content of the mixes decreased with increasing LS content. At 20% LS replacement, a slight reduction in air content (by ~0.4%) was observed due to the presence of cenospheres. However, at higher LS dosages (40–60%), the use of superplasticizers (SPs) to maintain slump led to a minor increase in air content. Overall, all mixes remained within the design range of 2 ± 0.5%, confirming that LS can be incorporated without compromising entrained air specifications.

The compaction factor improved with LS inclusion, particularly at higher replacement levels. For example, 40% and 60% of LS mixes showed an increase in compaction factors by 2.5% and 3.2%, respectively, compared to the control. This improvement was attributed to the leaner mix design and the use of SPs, which reduced internal friction during flow and placement. In contrast, fly ash mixes showed declining compactibility beyond 20% replacement, indicating LS’s superior compatibility in high-volume SCM systems.

Water penetration capacity (measured as water retention capacity—WRC) initially increased at 20% LS due to the water-absorbing cenospheres in LS. However, at higher replacements (40–60%), WRC declined, indicating reduced water-holding ability, likely due to increased flaky and irregular LS particles that introduced internal pores and reduced particle packing. This effect, while reducing surface water retention, did not negatively impact workability thanks to optimized SP dosage.

The fresh density of the concrete decreased proportionally with LS content. Compared to the control mix (~2436 kg/m^3^), mixes with 20–60% LS showed density reductions of 1.2–3.3%, due to the lower specific gravity of LS (2.46) compared to Portland cement (3.10). Nonetheless, all mixes remained within the typical fresh concrete density range (2200–2600 kg/m^3^), confirming LS’s suitability for producing standard-density concrete.

Although setting time was not directly measured for LS concrete in the same scope as other fresh properties, the paper notes that LS has a moderate pH (11.28) and high electrical conductivity, which implies early ion dissolution and faster initial hydration reactions compared to fly ash. These physicochemical properties suggest that LS may slightly accelerate early setting, especially when combined with superplasticizers and controlled mix temperatures.

**Figure 8:**
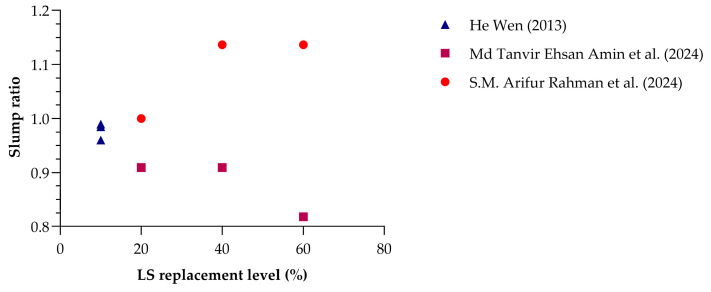
Slump ratio variation with respect to LS dosage across various studies [24,27,28].

Figure 8 compares the slump ratio (defined as the ratio of the slump of LS concrete to that of control concrete) at varying LS replacement levels across three independent studies [24,27,28]. The trends reveal notable differences among the studies, demonstrating that the effect of LS on fresh concrete workability is highly dependent on the mixed design, particularly the use of chemical admixtures and the physical nature of LS.

A study [20] reported relatively stable slump ratios (about 0.97–0.99) at low LS contents (5–10%), indicating negligible impact on workability at these levels without significant mix adjustments. In the study [28], a progressive decline in slump ratio was observed with an increase in LS dosage, reaching 0.81 at 60% LS. This clearly shows that higher LS content led to reduced workability, likely due to LS’s finer particles and irregular shapes increasing water demand. Importantly, the researchers noted that the amount of superplasticizer had to be increased in proportion to LS content, confirming that LS itself had a slump-reducing effect. A similar trend was reported by [27], where the slump ratio increased with LS dosage (up to 1.14 at 60%). However, this apparent improvement in workability was achieved through a corresponding increase in superplasticizer dosage, alongside the use of LS with cenosphere characteristics (hollow, spherical particles), which can naturally improve flow. Despite the increase in slump, the underlying data suggest that without the elevated SP dosage, workability would have declined, aligning with the physical behavior of high-volume LS inclusion.

Taken together, these studies suggest that while LS incorporation often reduces slump, this effect can be mitigated or even reversed through careful adjustment of admixture dosage and mix design parameters. However, the consistent need for an increased superplasticizer confirms LS’s inherent tendency to lower workability due to its high surface area, angularity, and water absorption capacity.

The observed reduction in slump with increasing LS content is primarily attributed to the angular and porous morphology of LS particles, which increases water demand and interparticle friction [27,42]. Moreover, due to the high surface area, LS affects the thickness of the water film around the cementitious particles, contributing to reduced flow [28,41]. Zeta potential measurements suggest that surface charge alterations in high-pH environments may impact particle dispersion and flocculation, further influencing rheological behavior [53]. These factors collectively explain the higher superplasticizer demand typically observed in LS-modified concrete [56].

### 4.3. Effect of LS Replacement Level and Curing Age on Compressive Strength Ratio

**Figure 9:**
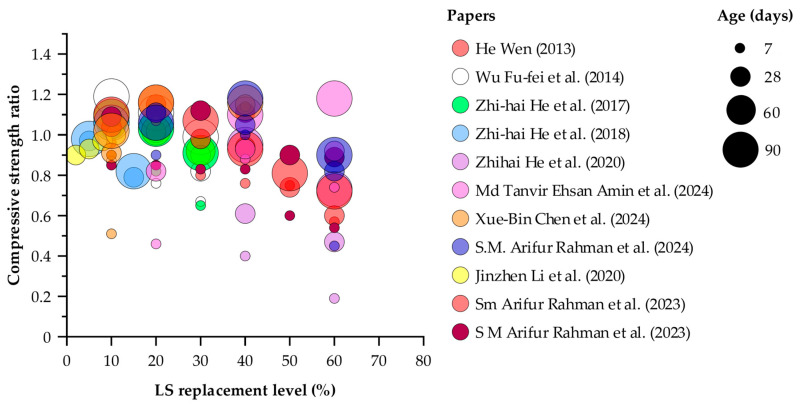
Normalized compressive strength ratio plotted against LS replacement percentage [2,7,24,27,28,29,32,41,44,61,64].

Figure 9 plots the compressive strength ratio (measured compressive strength divided by control strength) against LS replacement level for curing ages of 7, 28, 60, and 90 days. At seven days, the compressive strength ratio spans from 0.19 to 1.14, showing that early-age LS substitution can both markedly weaken and modestly strengthen concrete. By 28 days, ratios range from 0.47 to 1.15, with most tests achieving a value of at least 1.00, indicating that four weeks of curing generally restores or enhances compressive performance. At 60 days, the minimum observed ratio is 1.02 and the maximum is 1.18, demonstrating that ongoing pozzolanic reactions lead to consistent strength beyond the control mix. After 90 days, the ratios lie between 0.73 and 1.19, with the bulk value falling from 0.90 to 1.10; this confirms that moderate LS replacement can match or exceed reference strength over time, whereas substitution levels above 40 percent remain below unity even after extended curing. These results identify 40 percent as a practical upper limit for LS dosage under the tested conditions.

### 4.4. Effect of Concrete Strength Class on the Performance of Lithium Slag (LS) Blends

**Figure 10:**
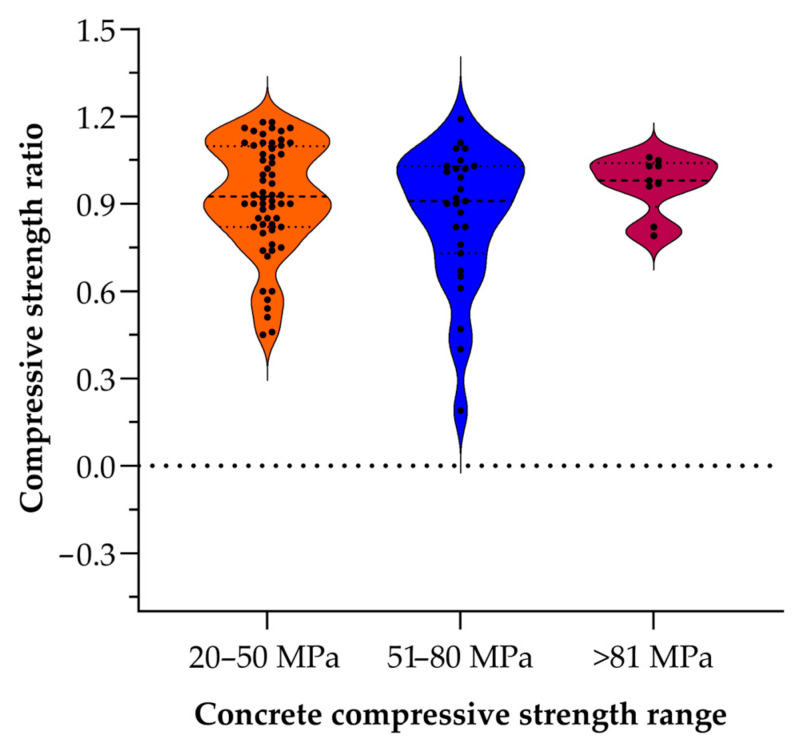
Distribution of normalized compressive strength ratios across three concrete strength classes (20–50, 51–80, >81 MPa) [2,7,24,27,28,29,32,41,44,61,64].

Figure 10 presents violin-and-dot plots of the compressive strength ratio—measured strength divided by reference strength—for three concrete classes: 20–50 MPa (orange), 51–80 MPa (blue), and >81 MPa (purple). The width of each violin reflects the relative frequency of observations, points show individual tests, and dashed lines mark the median and interquartile range. In the 20–50 MPa group, the ratios cluster tightly around a median of almost 0.93 (most between 0.6 and 1.2), indicating acceptable consistent behavior under the tested condition. The 51–80 MPa mixes display the greatest spread—from near 0.2 up to approximately 1.25, with a median just above one—suggesting that mid-range strengths are most sensitive to exposure. The >81 MPa category appears narrowest (roughly 0.8–1.1) with a median close to unity, implying high-strength concretes retain capacity most uniformly; however, this group is based on substantially fewer data points than the others. The limited sample size for the >81 MPa mixes may understate true variability and reduce confidence in the observed distribution, so these results should be interpreted with caution. Future work should expand the number of high-strength specimens to verify that their apparent stability is representative and not an artifact of a low sample count.

### 4.5. Effect of LS Replacement Level and Curing Age on Tensile Strength Ratio

**Figure 11:**
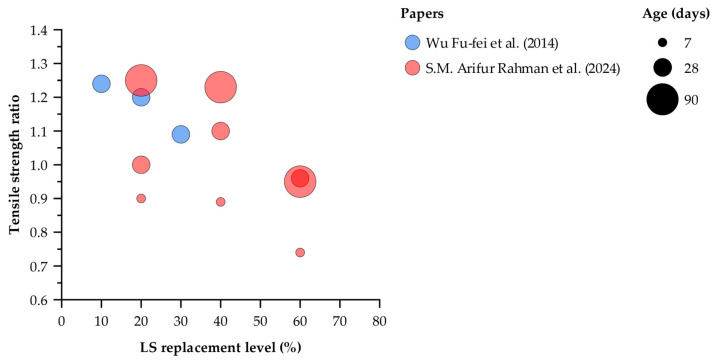
Tensile strength retention ratio (measured tensile strength divided by control strength) plotted against LS substitution percentage (10–60%) for curing durations of 7, 28, and 90 days [27,44].

Figure 11 shows the tensile strength ratio as a function of LS replacement level (10, 20, 30, 40, and 60%) after curing for 7, 28, and 90 days. At 28 days, mixes with 10% LS exhibit the highest ratio (1.24), followed by 20% (1.20) and 30% (1.09), indicating that modest substitutions not only restore but can exceed the control tensile capacity by four weeks. For the 20% replacement series, the ratio increases from 0.90 at 7 days to exactly 1.00 at 28 days and then to 1.25 at 90 days, demonstrating a sustained gain due to ongoing pozzolanic reactions. A similar trend is observed at 40% LS: the ratio rises from 0.89 at 7 days to 1.10 at 28 days and reaches 1.23 at 90 days, confirming that moderate dosages benefit progressively from prolonged curing.

In contrast, the 60% replacement mix starts with a low early-age ratio of 0.74, recovers to 0.96 by 28 days, but then slightly declines to 0.95 at 90 days. This behavior highlights a dilution effect at high LS contents, where the initial loss in tensile capacity cannot be fully compensated for even after extended hydration. Taken together, these data identify a practical upper limit of approximately 40% LS for achieving both early-age and long-term tensile performance; beyond this threshold, further substitution yields diminishing returns despite prolonged curing.

### 4.6. Effect of LS Replacement Level and Curing Age on Flexural Strength Ratio

**Figure 12:**
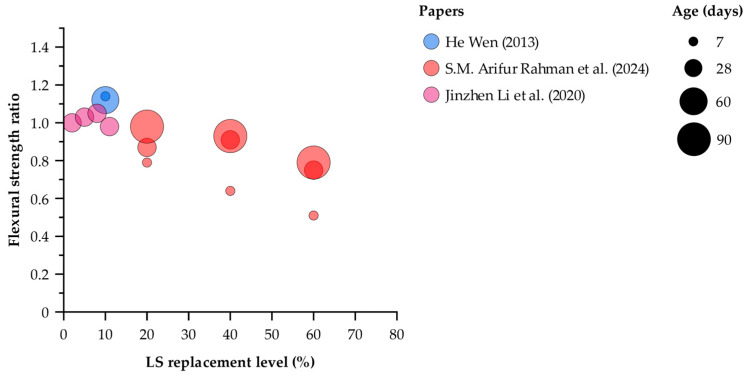
Flexural strength ratio (measured flexural strength divided by control strength) versus LS replacement level (%) after curing for 7, 28, 60, and 90 days [24,27,32].

Figure 12 shows that at very low LS contents (2–11%), the 28-day flexural strength ratios remain essentially unchanged or slightly improved (0.98–1.05), indicating that minor LS substitutions do not compromise flexural capacity. At 10% replacement, early-age performance is excellent, with a 7-day ratio of 1.14 and a 60-day ratio of 1.12, confirming both early and sustained enhancements. When LS content increases to 20%, the ratio rises steadily from 0.79 at 7 days to 0.87 at 28 days and ultimately approaches unity (0.98) at 90 days, reflecting progressive pozzolanic development. A 40% substitution follows a similar trajectory—0.64 at 7 days, 0.91 at 28 days, and 0.93 at 90 days—demonstrating that moderate dosages can recover most of the lost strength by three months. In contrast, the 60% mix suffers a severe dilution effect (0.51 at 7 days), partially recovers to 0.75 at 28 days, but remains below control strength (0.79) even after 90 days. These results indicate that while LS replacements up to about 40% can ultimately achieve near-control flexural performance with sufficient curing, higher substitution levels produce permanent strength deficits under the tested conditions.

### 4.7. Impact and Wear Resistance of LS-Based Concrete

The influence of lithium slag (LS) on the impact and abrasion performance of white reactive powder concrete (WRPC) has been examined in a recent study [32]. Their experimental program incorporated LS at varying replacement levels (0–11% by cement weight) to assess its effect on mechanical durability. Impact resistance, tested in 28 days using a standardized drop weight method, showed a notable enhancement at 5% LS content. Beyond this level, performance declined, likely due to changes in matrix toughness and microcrack propagation behavior. The improved resistance at lower LS levels was associated with enhanced interfacial bonding and densification, which allowed the material to absorb impact energy better. However, the decline at higher LS content (e.g., 11%) was attributed to increased brittleness, evidenced by a higher compressive-to-flexural strength ratio.

Wear resistance was evaluated by subjecting cube specimens to controlled surface abrasion under a fixed load. The results revealed that LS effectively enhanced abrasion resistance, with the mix containing 11% LS showing approximately half the wear loss compared to the control. This was largely credited to the filler and pozzolanic effects of LS, which refined the pore structure and improved the bond between aggregate and paste. Collectively, these findings suggest that incorporating LS in the range of 5–8% can simultaneously improve both impact and wear resistance in WRPC, making it a viable option for structural and architectural applications requiring high durability under mechanical stress.

### 4.8. Chemical Activation and Harsh Environment Performance of LS Concrete

Recent advances in lithium slag (LS)-based binders have highlighted the material’s potential not only as a sustainable cement substitute but also as a contributor to durability performance and enhanced reactivity in aggressive environments. Several studies have explored various strategies to optimize these properties through material design, chemical activation, and environmental exposure conditioning.

A study [53] investigated the freeze–thaw and sulfate resistance of recycled aggregate concrete incorporating LS. Their results revealed that a combination of 30% recycled coarse aggregate and 20% LS yielded the best resistance against cyclic deterioration. Indicators such as mass-change rate, relative dynamic modulus of elasticity (RDME), and residual compressive strength showed that moderate LS dosage significantly enhanced durability. Furthermore, they developed a damage prediction model based on RDME and compressive strength, validated through SEM analysis. However, mixes with excessive LS content displayed increased susceptibility to sulfate-induced degradation.

The role of chemical activation in improving LS reactivity was examined by the authors of Ref. [34], who used NaOH to activate LS in a blended cementitious system. They found that increasing the NaOH content (up to 6 wt%) led to an enhanced pozzolanic reaction, as evidenced by the increase in unconfined compressive strength (UCS) from early to late curing ages. At 28 and 56 days, the compressive strengths of the optimally activated system (CPL6.0) reached 32.3 MPa and 39.7 MPa, respectively, approaching values obtained with traditional Portland pozzolana cement. These improvements were corroborated by TGA and SEM analysis, which revealed a higher formation of C–S–H gel and a reduction in calcium hydroxide, indicating more complete hydration and improved microstructural density.

A study [17] focused on the corrosion behavior of LS concrete under simulated acid rain exposure. Their study demonstrated that LS improves resistance to acid-induced degradation, leading to lower compressive strength loss and reduced mass loss during wet–dry corrosion cycles. LS-enhanced concrete exhibited superior surface integrity and microstructural compactness. Hydration product analysis revealed that LS reduces CH crystal formation and increases C–S–H gel formation, lowering the Ca/Si ratio. However, a slight increase in neutralization depth was observed, likely due to the acid sensitivity of the matrix at high LS content.

In a more advanced application, the authors of [55] synthesized LS-based one-part geopolymers using hybrid solid activators (e.g., NaOH + Ca(OH)_2_, NaOH + CaCO_3_, NaOH + Na_2_SiO_3_). These activators not only improved mechanical strength—reaching up to 35.6 MPa at 28 days—but also refined pore structure and promoted the formation of C–A–S–H gels. Among them, CaCO_3_-based activators provided the best balance between activation efficiency and environmental compatibility, offering a low-alkalinity, cost-effective route for geopolymer production.

Together, these studies demonstrate that LS’s durability and reactivity can be significantly enhanced through targeted strategies, such as moderate dosage optimization, alkali activation, blending with recycled aggregates, and utilization in hybrid-activated systems. Such interventions not only unlock LS’s latent pozzolanic potential but also contribute to sustainable construction solutions in chemically and climatically aggressive environments.

**Figure 13:**
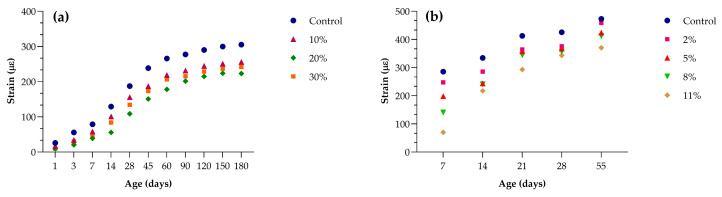
Drying shrinkage strain versus age for LS-substituted concretes at various replacement levels: data used from (**a**) Zhi-hai He et al. (2017) [61] mixtures with 0%, 10%, 20%, and 30% LS; (**b**) Jinzhen Li et al. (2020) [32] mixtures with 0%, 2%, 5%, 8%, and 11% LS.

Figure 13a shows that the control mixture undergoes progressive shrinkage, increasing from about 30 µε at 1 day to roughly 310 µε at 180 days. Incorporating 10% LS reduces long-term strain by approximately 10% (to almost 280 µε), while 20% and 30% substitutions lower it further to ~230 µε and ~240 µε, respectively. The greatest mitigation occurs between 7 and 90 days, where the 20% blend diverges most sharply from the control.

Figure 13b shows that the control reaches nearly 480 µε by day 55. Even a 2% LS addition cuts shrinkage to about 450 µε; higher dosages of 5%, 8%, and 11% progressively reduce it to approximately 425 µε, 400 µε. and 375 µε, respectively. All LS-containing mixes exhibit a similar curvature of strain versus age, but the absolute magnitude of shrinkage decreases with increasing LS content. These data demonstrate that moderate LS replacement (around 20–30%) offers the most effective long-term shrinkage control, while even small dosages (2–5%) deliver measurable benefits.

### 4.9. High-Volume Lithium Slag Composite Concrete

The study [28] provides a comprehensive evaluation of concrete incorporating high volumes of lithium slag (LS) as a supplementary cementitious material (SCM), with replacement levels of 20%, 40%, and 60% by weight of cement. The investigation focused on the effects of LS on compressive strength development, transport properties, porosity, and microstructural characteristics over extended curing durations of 28, 90, and 180 days. The findings revealed that incorporating LS at up to a 40% replacement level led to marked improvements in long-term mechanical and durability performance. While early-age compressive strength decreased due to LS’s slower pozzolanic activity and limited calcium hydroxide availability, the long-term strength gain was significant. At 180 days, the 40% LS mix achieved a 18.3% higher compressive strength than the control, a result of continued hydration and the formation of additional calcium silicate hydrate (C–S–H) gel promoted by the reactive silica and alumina present in LS.

**Figure 14:**
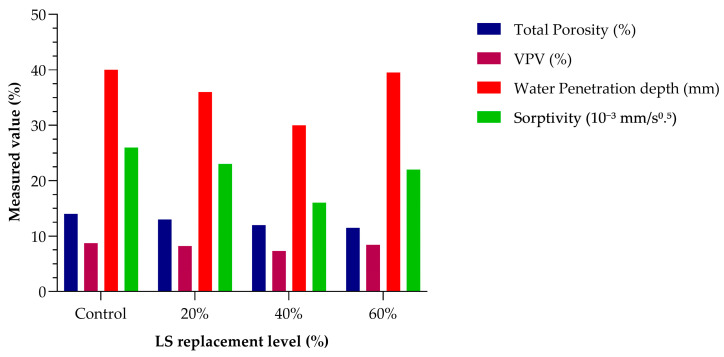
Durability-related properties of LS concrete at 180 days. Data used from [24].

In addition to mechanical performance, the 40% LS mix exhibited the most favorable transport-related properties. At 180 days, it showed a 31.3% reduction in the volume of permeable voids (VPV), a 36% reduction in water penetration depth, and a 75.7% decrease in sorptivity relative to the control mix. These enhancements are attributed to a denser pore structure, as confirmed through porosity testing and SEM analysis. Notably, the macro-porosity of the 40% LS mix was reduced to just 1.7%, the lowest among all the tested compositions. SEM and EDS analyses also indicated the formation of secondary products such as CaCO_3_ and ettringite, which contributed to additional pore refinement and matrix densification. These quantitative results are illustrated in Figure 14, which compares the key durability-related parameters total porosity, VPV, water penetration depth, and sorptivity across different LS replacement levels. As shown, all indicators of permeability and porosity improved substantially with 40% LS incorporation, supporting the claim of enhanced durability. In contrast, the 60% LS mix displayed a reversal in performance trends, with increased porosity, more unreacted particles, and reduced strength, likely due to insufficient calcium hydroxide for full LS activation. This study highlights the potential for using high-volume LS as a viable SCM. A replacement level of around 40% offers an optimal balance between strength development, permeability reduction, and pore structure refinement, making LS a promising material for durable and environmentally resilient concrete applications.

### 4.10. High-Temperature Performance of LS-Based Concrete

The thermal behavior of lithium slag concrete (LSC) was studied by the authors of [47], who investigated the residual mechanical properties of LSC exposed to elevated temperatures ranging from 100 °C to 700 °C. Their experimental program examined three key mechanical indicators—cube compressive strength, prism compressive strength, and flexural strength—under two cooling regimes, natural cooling (NC) and water spray cooling (WSC), across four LS replacement levels (0%, 10%, 20%, and 30%). The results revealed that moderate temperatures (up to 100 °C) caused minimal strength reduction and even slight improvements in specific cases. For example, specimens with 20% LS exhibited an approximately 8% increase in prism compressive strength, attributed to continued hydration and densification facilitated by water vapor entrapment. In contrast, temperatures above 300 °C led to severe degradation, with concrete losing up to 80% of its compressive strength at 700 °C, due to decomposition of C–S–H, dehydration of CH, and decarbonation of CaCO_3_.

Among the tested mixtures, 20% LS replacement consistently exhibited the best performance across all mechanical parameters at elevated temperatures. It not only minimized mass loss but also retained higher residual strengths compared to mixes with 0%, 10%, or 30% LS. At 700 °C, the flexural strength of LSC with 20% LS was approximately 50% higher than that of ordinary concrete, indicating LS’s beneficial role in delaying structural degradation under thermal stress. Interestingly, the cooling method (NC vs. WSC) had a limited impact on mechanical properties when the WSC duration was short (3–5 min). Although the literature often highlights WSC as detrimental due to thermal shock, this study found that short-duration WSC resulted in only slightly lower strengths than NC, suggesting that LS-modified concrete may tolerate rapid post-fire cooling better than conventional mixes. Mass loss trends support these findings. LSC with 20% LS showed the lowest weight reduction after thermal exposure, due to its dense internal microstructure formed by efficient pozzolanic reactions between SiO_2_ (from LS) and Ca(OH)_2_ (from cement). This densification limited both water evaporation and structural damage.

### 4.11. Durability and Long-Term Performance

Various durability indicators were investigated in LS-blended systems, including chloride ion penetration, acid resistance, and freeze–thaw cycling performance. To allow a more meaningful comparison, these metrics are reviewed together using LS replacement level (typically 10–40%) as a baseline axis. For example, concretes incorporating 20–30% LS consistently exhibited 25–40% reductions in chloride ion penetration depth compared to plain cementitious systems [27,28] attributed to denser C–S–H gel formation and reduced capillary pore connectivity. In acid environments, mass loss reduced by 15–30% at optimal LS content, while over-replacement reversed these gains [49]. Freeze–thaw durability also improved, with specimens retaining up to 90–95% of their original compressive strength after 300 cycles when blended with 20–25% LS [57]. These findings demonstrate a converging trend: moderate LS dosages consistently yield superior durability performance. However, a lack of harmonized test methods, curing protocols, and reporting standards across studies makes direct quantitative comparison challenging. It is recommended that future work adopt standardized multi-metric durability frameworks to improve reproducibility and industrial translation.

### 4.12. Creep Behavior of LS-Modified Concretes

**Figure 15:**
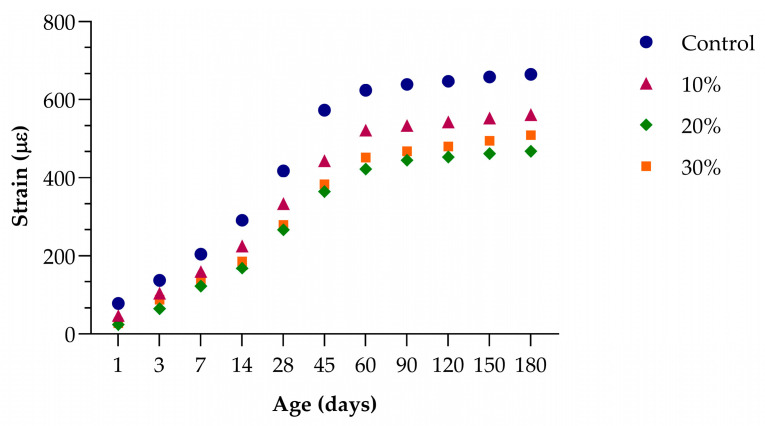
Development of creep strain under sustained loading for concrete containing 0%, 10%, 20%, and 30% LS over 180 days. Data used from [52].

Figure 15 shows that incorporating lithium slag (LS) significantly reduces long-term creep strain in concrete in a clear, dose-dependent manner over a period of 180 days. At an early age, the control mix exhibits the highest initial creep strain, while concretes containing LS demonstrate an immediate reduction of approximately 40% to 70%, depending on the replacement level. As curing continues, this trend remains consistent. Within the first week, the control mix develops nearly twice as much creep strain as the concrete with a 20% LS replacement, while the 10% and 30% mixes show intermediate performance with noticeable reductions compared to the control. During the primary creep phase, which extends up to about two months, the control concrete shows the steepest increase in deformation, whereas the mixes containing LS maintain significantly lower creep values—generally around 20% to 30% less than the control.

After about three months, the creep strains tend to stabilize. By 180 days, the control mix reaches its maximum strain, while concretes with LS maintain lower final values. Notably, the mix with 20% LS consistently shows the greatest reduction, achieving almost one-third lower long-term creep than the plain concrete. The 10% and 30% substitutions also provide meaningful mitigation of time-dependent deformation, though the effect is slightly less pronounced than with the 20% replacement. These results confirm that partial replacement of cement with moderate amounts of LS can effectively reduce creep in concrete under sustained loading, thereby enhancing long-term dimensional stability.

### 4.13. Application of Lithium Slag in Autoclaved Aerated Concrete (AAC)

Autoclaved aerated concrete (AAC) incorporating lithium slag (LS) was produced by replacing quartz sand (QS) using two distinct strategies: (1) “effective element control,” in which the Ca/Si ratio of the mix was held constant, and (2) “material quality control,” which maintained a fixed siliceous mass. In both methods, LS was blended with OPC, lime, gypsum, aluminum powder (0.1% by mass), and calcium stearate (1%), cast into 70.7 mm cubes, pre-cured at 60 °C and 50% RH for 6 h to promote aeration, and then autoclaved at 190 °C under 12 bars for 10 h before oven-drying to constant weight [30]. Under the material quality control approach, partial replacement of QS with LS (typically up to 20–30%) led to a notable increase in compressive strength, with reported values exceeding those of the control mix by approximately 15–20%. Simultaneously, the bulk density decreased by around 8–12%, a result attributed to LS’s high pozzolanic activity under hydrothermal conditions. The reactive silica–alumina phases in LS promote enhanced formation of secondary C–S–H and C–A–S–H gels, which densify the interfacial transition zone. However, when LS content exceeded 40–50%, the increased slurry viscosity impaired pore expansion during pre-curing and interfered with hydration during autoclaving, ultimately reducing mechanical strength [30]. Thermal conductivity testing showed that mixes with optimal LS dosage (20–30%) exhibited lower thermal conductivity values (typically reduced by 10–15%), attributed to a more refined and uniform pore structure that inhibited heat transfer. Over-replacement, on the other hand, led to denser matrices with elevated conductivity, in direct correlation with increased bulk density. Water absorption tests similarly demonstrated that well-balanced LS substitution reduced capillary uptake by up to 25%, owing to decreased macropore connectivity. In contrast, high LS dosages increased permeability [30]. Microstructural characterization revealed strong compositional and morphological differences. XRD patterns confirmed the presence of residual crystalline phases (quartz, muscovite, spodumene), hydrothermal products (tobermorite, xonotlite, katoite), and carbonation products (calcite). Attenuation of the 11.3 Å tobermorite peak and enhancement at 3.5 Å indicated Al^3+^ substitution for Si^4+^ in the silicate chains. FTIR spectra supported these findings, showing intensified Si–O bending/stretching bands at moderate LS dosages, while excessive Al substitution hindered gel polymerization. SEM–EDS imaging revealed a morphological shift from plate-like tobermorite in the control mix to needle-like tobermorite and katoite in LS-based mixes. Atomic-scale analysis showed a linear increase in Al content in hydration products proportional to LS dosage [30]. These quantitative and microstructural results demonstrate that, when carefully dosed and processed, LS can function as an effective siliceous replacement in AAC, improving compressive strength, reducing density and thermal conductivity, and enhancing pore structure. Nonetheless, the findings also highlight the importance of establishing standardized mix design parameters and processing protocols to ensure reproducible outcomes at scale.

### 4.14. Comparative Overview of Experimental Studies on Lithium Slag-Modified Concrete

To better contextualize the experimental findings and performance characteristics of lithium slag (LS) in various concrete systems, Table 1 provides a comparative overview of notable studies conducted over the past decade. The table highlights the type of concrete investigated, the replacement levels of LS, the testing protocols employed (e.g., strength, durability, microstructural analysis), and the optimal dosages reported. This summary underscores the versatility of LS across different concrete types—from normal strength to ultra-high-performance concretes—and its influence on mechanical performance, durability enhancement, and microstructure refinement.

### 4.15. Comparative Performance of LS from Different Ore Sources

Numerous studies have shown that the performance of lithium slag (LS) in cementitious systems is highly dependent on its mineral origin, particularly whether it is derived from lepidolite or spodumene ores. However, limited comparative analyses have been conducted across studies using standardized metrics. For instance, lepidolite-derived LS typically contains higher levels of alumina and potassium, which can influence the formation of secondary hydration products such as C–A–S–H gels, potentially enhancing long-term strength development. In contrast, spodumene-derived LS generally contains higher silica content and lower alkali content, which contributes to denser microstructures but may require additional activation or blending with other pozzolans to optimize performance [37,45]. Although direct cross-study comparisons are complicated by variations in mix designs and testing protocols, trends can be observed. For example, the compressive strength reported for concrete with 20% spodumene LS after 28 days was approximately 55 MPa [43], while mixes with lepidolite-derived LS at similar dosages reached comparable or slightly higher strengths (~58 MPa) under similar water/binder ratios [38]. These variations suggest differences in reactivity and hydration kinetics stemming from the underlying mineralogy. To facilitate broader conclusions, future research should adopt uniform testing methodologies and include chemical and mineralogical characterization of LS sources, enabling systematic comparisons and predictive modeling. Establishing such standardized benchmarks is essential to generalize LS performance and guide its industrial application.

### 4.16. Quantitative Gel-Phase Characterization

Although XRD, SEM, and FTIR analyses have confirmed the presence of secondary C–S–H and C–A–S–H gels in LS-modified concretes, quantitative phase measurements remain scarce. Techniques such as thermogravimetric analysis (TGA/DTG) and solid-state 29Si MAS-NMR are needed to resolve gel fractions and silicate network development. For example, Ref. [34] employed TGA/DTG to quantify bound-water loss in cemented paste backfill containing LS, correlating mass-loss peaks with C–S–H decomposition and overall gel content. Furthermore, Ref. [57] used TG-DSC and MIP to determine the gel yield and pore structure evolution in micro-lithium slag-accelerated cement mortars, demonstrating how different LS dosages influence hydration product distribution. Meanwhile, Ref. [8] applied 29Si NMR to alkali-activated LS pastes, identifying Q^0^–Q^4^ silicon environments and tracking the polymerization degree of N–A–S–H and C–A–S–H gels. Adopting these multi-technique approaches in ordinary and high-performance LS concretes would (1) precisely quantify gel fractions at various replacement levels, (2) compare reactivity between spodumene- and lepidolite-derived LS, and (3) link gel content directly to strength, permeability, and time-dependent deformation. It is, therefore, recommended that future LS concrete research routinely include TGA/DTG and 29Si NMR analyses to establish robust, quantitative microstructural benchmarks. To complement experimental data, thermodynamic and reactive transport modeling can elucidate the driving forces and sequence of hydration and pozzolanic reactions in LS-cement blends. For instance, Ref. [66] developed generalized solutions for advection–dispersion equations with time- and space-dependent sources, enabling the simulation of coupled transport and chemical reactions in porous media. Applying a similar approach to LS systems would involve (1) defining source terms for cement dissolution and LS pozzolanic reaction based on measured oxide compositions, (2) coupling these with transport equations for Ca^2+^, Si(OH)_4_, and Al species, and (3) using equilibrium thermodynamics to predict phase assemblages (C–S–H, C–A–S–H, ettringite) as a function of time and position. Such a framework could predict strength development kinetics by relating gel volume fractions to mechanical stiffness and explain spatial heterogeneities in microstructure and durability performance. We therefore recommend that future research on LS-modified concretes integrate reactive transport modeling—like the methodology of [66]—to provide a unified theoretical basis for observed experimental trends.

## 5. Conclusions

This review comprehensively explored the potential of lithium slag (LS) as a supplementary cementitious material (SCM) in concrete applications. Based on the analysis of the experimental findings across various studies, the following key conclusions are drawn:

(1) Optimal Replacement Level

Across the reviewed literature, a lithium slag (LS) replacement level in the range of 20–30% by mass of cement is most commonly associated with improved mechanical strength, durability, and dimensional stability. While specific results vary due to differences in mix design, curing conditions, and LS composition, this range appears to provide a favorable balance in most reported cases. Replacement levels beyond 40% often lead to diminishing returns, particularly in tensile and flexural performance. It is important to note, however, that these conclusions are based on general trends observed in published studies and are not derived from a unified statistical or multi-criteria decision analysis.

(2) Mechanical Properties

Moderate LS incorporations (10–30%) enhance long-term compressive strength by up to 25% compared to control mixes, with peak gains observed at 60–90 days due to pozzolanic activity. Tensile and flexural capacities likewise exceed control values at 28 and 90 days when LS is kept below 40%.

(3) Dimensional Stability (Shrinkage and Creep)

Drying shrinkage is reduced by up to 30%, and sustained-load creep strains decrease by 30–40% at 20–30% LS, demonstrating dose-dependent mitigation of time-dependent deformations. These improvements contribute to crack resistance and long-term serviceability.

(4) Fresh-State Behavior

The fine, angular particles of LS increase water demand and reduce workability, but careful adjustment of superplasticizer dosage fully restores slump and flow without compromising strength or durability.

(5) Durability in Aggressive Environments

LS-modified concretes exhibit enhanced resistance to sulfate attack, acid exposure, abrasion, freeze–thaw cycles, and chloride ingress. These gains are attributed to pore-structure refinement, increased C–S–H formation, and reduced permeability.

(6) High-Volume and High-Temperature Performance

At 40% replacement, 180-day compressive strength can improve by up to 18%, with sorptivity reductions exceeding 30%. Under elevated temperatures (up to 700 °C), 20% LS mixes retain up to 50% more residual strength than controls.

(7) Advanced Systems and Circular-Economy Applications

In advanced systems—including AAC, one-part geopolymers, and recycled aggregate concretes—LS promotes denser microstructures and enhances durability, highlighting its potential for use across a range of sustainable concrete technologies.

(8) While this review identified significant variability in LS composition, the lack of consistently reported chemical and performance data across studies prevented the development of a quantitative model linking composition to pozzolanic activity. Future research should prioritize standardized testing and multivariate analysis to better understand and predict the performance of LS-based cementitious systems.

## Figures and Tables

**Table 1:** Summary of the research conducted on LS concretes.

Ref.	Concrete Type	Level of the LS (By Weight of Cement)	Research Conducted	Optimum Dosage	Highlighted Results
[63]	High-performance lithium slag concrete	Ranging from 15% to 45% of the binder	Ring method test to evaluate early-age anti-cracking capability, splitting tensile strength, crack width, and cracking times.	30%	The crack width and number of cracks in the concrete specimens significantly decreased.
[24]	High-performance concrete (HPC)—80 MPa	10% to 40%	Compressive strength test, flexural strength test, and chloride ion penetration resistance test.	10%	(1) Good long-term strength development. (2) With high LS content, early and later strength development was negatively affected. (3) Improved resistance to chloride ion penetration.
[44]	High-performance concrete (HPC) with lithium slag and fly ash—70 MPa	0%, 10%, 20%, and 30%	Compressive strength, splitting tensile strength, early crack resistance, chloride ion permeability, and the microstructure.	Mechanical properties: 10%. Crack resistance and chloride ion permeability: 30% lithium slag and 20% fly ash	(1) Combining lithium slag with fly ash improved the concrete’s crack resistance and impermeability. (2) Improved both mechanical properties and durability.
[25]	High-Performance Concrete (HPC) with lithium slag and steel slag	67.5 kg/m^3^ to 175 kg/m^3^	Chloride ion diffusion coefficient.	The optimum dosage of lithium slag was 15% combined with 40% steel slag	(1) The chloride ion diffusion coefficient decreased as the water–binder ratio decreased. (2) A combination of lithium slag and steel slag reduced the chloride ion diffusion coefficient. (3) The combination of lithium slag and steel slag could replace up to 75% of cementitious materials in HPC.
[1]	Normal concrete	0%, 10%, 20%, and 30%	Drying shrinkage, carbonation resistance, wear resistance, and chloride ion penetration.	20–30%	(1) LS improved drying shrinkage. (2) Wear resistance initially increased with LS but declined when LS content exceeded 20%. (3) Chloride ion resistance improved significantly. (4) Enhanced concrete density and reduced formation of ettringite.
[61]	Normal concrete—50 MPa	0%, 10%, 20%, and 30%	Compressive strength, elastic modulus, drying shrinkage, and creep, mercury intrusion porosimetry (MIP) and scanning electron microscope (SEM) analyses.	20%	(1) Improved the compressive strength and elastic modulus at later ages. (2) Drying shrinkage and creep strain reduced. (3) Improved mechanical properties. (4) Excessive dosages (30%) led to reduced performance.
[2]	UHPC—150 MPa	5%, 10%, and 15%	Compressive strength, microstructure (using SEM and MIP), and pore structure.	10%	(1) Improved the compressive strength. (2) The microstructure of UHPC improved. (3) Higher contents of lithium slag (15%) negatively affected the pore structure and strength.
[60]	Sulphoaluminate cement paste	2.5%, 5.0%, and 10%	Compressive strength tests, setting time tests, conductivity, mercury intrusion porosimetry (MIP), XRD, SEM, and hydration heat analysis.	2.50%	(1) Wet-grinding lithium slag (WGLS) particles greatly improved the early hydration and strength. (2) The optimum dosage of 2.5% WGLS resulted in a 44.4% increase in 7-day compressive strength.
[59]	Normal concrete—50 MPa	75 kg/m^3^	The influence of polypropylene fibers, the number of thermal-cold cycles (0, 100, 300, and 500), and the flexural loading level (0.20 and 0.35 of the experimental peak moment). It measured cracking load, ultimate load, deflection, and crack width.	Not reported	(1) After 100 TC cycles, the flexural performance of the beams improved significantly but deteriorated slightly after 300 TC cycles. (2) The addition of PP fibers enhanced both the cracking load and the ultimate load. (3) Significant impact on the flexural properties of reinforced concrete beams.
[8]	Alkali-activated lithium slag geopolymer paste	The specific mix proportions are based on the molar ratio of SiO_2_/Al_2_O_3_ (4.53 to 5.60)	Compressive strength tests, isothermal calorimetry, X-ray diffraction (XRD), scanning electron microscopy (SEM), 29Si NMR, Fourier transform infrared (FT-IR) spectroscopy, and pore solution analysis (using ICP-OES).	Best performance in terms of compressive strength and microstructure was observed at an SiO_2_/Al_2_O_3_ molar ratio of 5.6.	(1) Better early-age compressive strength. (2) Enhanced the material’s strength. (3) Dissolution of lithium slag led to increased concentrations of silicon and aluminum, which supported the formation of geopolymeric gel.
[58]	Recycled coarse aggregate concrete—35 MPa	10%, 15%, 20%, and 25%	Cube compressive strength, axial compressive strength, splitting tensile strength, flexural strength, elastic modulus tests, and scanning electron microscope (SEM) analysis of the microstructure.	20%	Compressive strength increased by 17.36%, axial compressive strength by 17.44%, and splitting tensile strength by 46.48%.
[31]	Steam-cured Cement mortar	20%	Hydration product analysis (XRD), pore structure evaluation (MIP), compressive strength tests, and flexural strength tests. The study also involved long-term exposure to a 5 wt% sodium sulfate solution.	20%	(1) Improved the sulfate resistance of cement mortars. (2) Increased the proportion of gel pores and decreased the capillary pores, which contributed to better durability under sulfate attack.
[57]	Normal concrete—40 MPa Portland cement mortar with micro-lithium slag as an accelerator	Micro-lithium slag was used at levels of 0.5%, 1%, 2%, and 4%	Compressive strength tests, hydration heat analysis, SEM (Scanning Electron Microscopy), XRD (X-ray diffraction), TG-DTG analysis (thermogravimetric), and MIP (Mercury Intrusion Porosimetry).	4%	(1) Significantly increased the early-age compressive strength. (2) Accelerated hydration and refined the pore structure.
[64]	Normal concrete—50 MPa	40% and 60%	Compressive strength tests, non-evaporable water content analysis, scanning electron microscopy (SEM), and mercury intrusion porosimetry (MIP)	40%	(1) Reduced the early-age compressive strength, but the strength improved significantly at later ages. (2) The 60% lithium slag mix had increased porosity and lower compressive strength due to incomplete pozzolanic reactions. (3) SEM and MIP analysis showed that 40% lithium slag refined the pore structure and improved the microstructure of the concrete.
[32]	White reactive powder concrete (WRPC)	2%, 5%, 8%, and 11%	Flexural strength, wear resistance, drying shrinkage, chloride migration, microstructure analysis (SEM), and hydration analysis (TG-DSC).	8% lithium slag, improving the flexural strength. 11% lithium slag, reducing wear by dry shrinkage and electric flux.	Adding 8% lithium slag improved the flexural strength and wear resistance of WRPC. Lithium slag content of 11% showed significant reductions in dry shrinkage and chloride penetration while maintaining whiteness similar to the control.
[62]	Recycled concrete with lithium slag—35 MPa	At levels of 20%	Freeze–thaw cycles and sulfate freeze-thaw coupling cycle tests, relative dynamic modulus of elasticity (RDME), and compressive strength, scanning electron microscopy (SEM).	20% lithium slag combined with 30% recycled coarse aggregate	(1) 30% RCA and 20% lithium slag provided optimal durability, with improved resistance to freeze-thaw cycles and sulfate attack. (2) Increased susceptibility to sulfate erosion.
[34]	Cemented paste backfill (CPB)—30 MPa	30%	Unconfined compressive strength (UCS) tests, X-ray diffraction (XRD) analysis, thermo-gravimetric analysis (TGA), and scanning electron microscopy (SEM).	30%	Significantly improved the pozzolanic activity of lithium slag.
[10]	Lightweight aggregate—up to 9 MPa	10%, 20%, 30%, and 40%	Bloating index (BI), particle density, single pellet compressive strength, water absorption, X-ray diffraction (XRD), and SEM analysis.	30%	(1) Improved the expansion properties of the lightweight aggregate while reducing the sintering temperature and energy consumption. (2) Showed good mechanical properties and met the required standards for lightweight aggregates.
[21]	Normal concrete—60 MPa	20% and 40%	Compressive strength tests, mass loss measurements, neutralization depth analysis, and XRD, SEM, TG/DTG, and EDS analyses.	40%	(1) Highest acid rain resistance, with better appearance integrity, a lower mass loss rate, and the highest residual compressive strength after 100 days. (2) Improved the compactness of the microstructure, reducing the Ca/Si ratio of the C-S-H gel, which contributed to better acid resistance. (3) The neutralization depth of concrete increased with higher lithium slag content, indicating a potential downside to using large amounts of lithium slag in acidic environments.
[65]	Normal concrete—45 MPa	10–20%	Mechanical, microstructural, and durability properties.	10–20%	(1) The use of 10–20% LRR showed improved mechanical properties. (2) Enhanced resistance to chloride migration, sulfate attack, and shrinkage, promoting better durability.
[20]	Alkali-activated material (AAM)—16.9 MPa to 49.9 MPa.	Lithium slag was used to replace metakaolin at levels of 25%, 50%, 75%, and 100%	The research aimed to investigate the effect of lithium slag (LS) replacing metakaolin (MK) in alkali-activated materials. The study examined rheology, initial fluidity, setting time, compressive strength, phase composition, and microstructure. It also included cost and energy consumption analysis.	25% to 50% lithium slag	(1) The compressive strength increased. (2) Improved the fluidity of alkali-activated metakaolin-slag pastes. (3) Improved the pore structure.
[56]	Lithium slag–steel slag-based cement—45 MPa	5%, 10%, and 20%	Workability, compressive strength, and microstructural characteristics.	0% lithium slag and 10% steel slag (L10S10)	(1) The highest compressive strength recorded was 48.2 MPa at 28 days for the L10S10 mix (10% LS, 10% SS). Increasing the percentage of steel slag beyond 10% had a negative impact on strength. (2) Fluidity decreased with higher percentages of LS, while SS improved fluidity. (3) The mix with 10% LS showed improved microstructure.
[55]	Lithium slag-based geopolymer synthesized using hybrid solid activators—35 MPa	Lithium slag was used with 5% hybrid activators	Workability, mechanical properties (including compressive strength), hydration process, microstructure, and pore structure.	Not reported	(1) The highest compressive strength recorded was 35.6 MPa at 28 days for the geopolymer using NaOH + CaCO_3_ as the hybrid activator. (2) The fluidity of the geopolymer mixes was about 190 mm, with no significant difference between the hybrid activators. (3) The combination of Ca(OH)_2_ and CaCO_3_ resulted in a higher hydration rate and more C-A-S-H gel formation. (4) The hybrid activator containing CaCO_3_ resulted in a denser pore structure.
[51]	Ultra-high performance concrete (UHPC) with compressive strengths ranging higher than 150 MPa	At levels of 30%, with various proportions of limestone powder (LP) incorporated (5%, 10%, and 20%).	At levels of 30%, with various proportions of limestone powder (LP) incorporated (5%, 10%, and 20%).	10% limestone powder (LP) and 20% lithium slag (LS)	(1) Increased compressive strength. (2) The addition of limestone powder improved the fluidity of UHPC, with the mix containing 10% LP showing the best balance of workability and strength. (3) Incorporating limestone powder refined the pore structure, contributing to improved durability and reduced porosity.
[50]	Portland pozzolana cement (PPC) blended with modified lithium slag (MGLS)	30% lithium slag modified with 0-15% MgO.	Investigates the pozzolanic activity of lithium slag (LS) modified with MgO through a melting-quenching-grinding process. The study examines the physical and chemical properties, unconfined compressive strength (UCS), and microstructure of PPC blended with modified lithium slag (MGLS).	The mix with 15% MgO-modified lithium slag	(1) Higher strength at 90 days. (2) MgO modification enhanced the pozzolanic activity of lithium slag, promoting the formation of pozzolanic products (CSH and CAH gels). (3) Improved the compactness of the microstructure and increased the Ca/Si and Al/Si ratios in the CSH/CAH gels, further enhancing strength and durability.
[7]	Normal concretes—50 MPa	Lithium slag was used at levels of 0–60%	Investigates the pozzolanic activity and microstructural development of lithium slag as a supplementary cementitious material. It involved various tests like the Frattini test, strength activity index (SAI) test, and R3 test to assess the impact of lithium slag replacement on compressive strength, microstructure, and hydration products.	40% lithium slag	(1) Increased compressive strength. (2) Promoted the formation of ettringite, monocarboaluminate, and calcium aluminosilicate hydrates (C-S-H), improving the microstructure. (3) Showed significant pozzolanic activity, as confirmed by the Frattini and R3 tests.
[52]	Geopolymer concrete—12.8 MPa to 52.2 MPa	10% to 100% LS as a partial or full replacement in geopolymer concrete mixtures.	Workability, strength, durability, and microstructure of LS-based geopolymers.	Around 20–50% LS can improve strength and durability performance, with the best compressive strength recorded for mixes with 50% LS combined with GGBFS.	(1) Increased compressive strength. (2) Incorporating ground granulated blast furnace slag (GGBFS) alongside LS improves the fluidity and setting times of geopolymer mixtures. (3) LS geopolymers exhibit enhanced resistance to sulfate attack and reduced drying shrinkage when combined with GGBFS.
[54]	Normal concrete—50 MPa	The study used a ternary system with 33% of lithium slag (LS), iron ore tailings, and phosphate slag as cement replacements.	Explores the impact of different SCM dosages, particle size distributions, and the proportions of IOTs on compressive strength, matrix pore structure, and the interfacial transition zone (ITZ).	33% IOTs, phosphate slag, and lithium slag in the SCM	(1) Increased compressive strength. (2) Reduced cumulative pore volume and improved matrix pore structure. (3) Enhanced the ITZ by reducing porosity and promoting better bonding between the aggregate and the cement matrix.
[38]	Cementitious and geopolymer composites with lithium slag incorporation	Up to 30% lithium slag (LS)	LS’s physiochemical properties, mechanical strength, durability, and environmental impact compared to other SCMs such as fly ash (FA) and ground granulated blast furnace slag (GGBFS). The paper also discusses LS’s role in enhancing chloride ion resistance and reducing drying shrinkage.	LS content up to 30%	(1) Enhanced compressive strength, but further increases led to diminishing returns. (2) Improved chloride ion resistance and reduced drying shrinkage. However, its effect on setting time and flowability can vary based on dosage.
[53]	Lithium slag–cement binder with sodium sulfate admixture—45 MPa	20%	Investigates the rheological performance and hydration kinetics of a cement binder blended with lithium slag (LS) and sodium sulfate. It examines how sodium sulfate affects the viscosity, hydration, and mechanical properties of the lithium slag–cement binder by analyzing microstructure, setting time, and hydration heat.	20% lithium slag was used, and the best performance was obtained with 0.4% sodium sulfate	(1) With 20% LS and 0.4% sodium sulfate, the compressive strength increased compared to the control mix without sodium sulfate. (2) The addition of sodium sulfate accelerated the hydration process, enhancing the formation of calcium silicate hydrate (C-S-H) and ettringite (AFt), leading to a denser microstructure. (3) Increased viscosity and reduced flow behavior, indicating enhanced consistency in the binder.
[41]	Lithium slag-cement composites	0% to 60%	Workability, rheological behavior, hydration heat, air content, and compressive strength using different models and testing methods, including mini-slump tests and rheology models.	40%	(1) The mini-slump diameter decreased with increasing LS content, but the mix with 40% LS showed comparable workability to the control. (2) The hydration heat increased with LS content, and the 40% LS mix generated 300 J/g of exothermic heat at 72 h. (3) The shear stress for the 40% LS paste was similar to that of the control, while pastes with more than 40% LS became more viscous.
[49]	Geopolymer concrete—45 MPa	The study replaced 0%, 10%, 20%, 30%, and 40% of lithium slag with silica fume	Compressive strength, residual strength after exposure to acidic environments (HCl and H_2_SO_4_), and microstructural analysis using SEM/EDS.	40% replacement of lithium slag with silica fume	(1) The compressive strength increased with silica fume content, with sodium-activated geopolymers showing higher strength compared to potassium-activated ones. (2) Geopolymers activated with sodium and potassium showed strength degradation when exposed to H_2_SO_4_ and HCl. The residual strength of specimens exposed to HCl was higher than that of those exposed to H_2_SO_4_. (3) SEM/EDS analysis revealed micro-cracking due to calcium sulfate formation in the LSG after exposure to H_2_SO_4_, and leaching of aluminum from the aluminosilicate gel upon exposure to HCl.
[48]	UHPC (140 MPa)–HPC (60 MPa)	10% to 20%	Reviews the management and reuse of lithium refinery residue (LRR) in China.	10% to 20%, depending on the application	(1) 10% LRR improves compressive strength and sulfate resistance in cement. (2) Incorporating up to 20% LRR enhances the mechanical properties of concrete, including compressive strength, elastic modulus, and shrinkage resistance. (3) Using LRR in geopolymer fabrication leads to significant increases in compressive strength with heat activation. (4) The sulfur and sodium content in LRR can pose challenges for reuse in construction materials, but processing techniques like activation or combining with other materials (fly ash, slag) can improve reactivity.
[29]	Lithium slag recycled fine aggregate concrete—30 MPa	0%, 10%, 20%, and 40%	The research examined the effects of lithium slag content (0%, 10%, 20%, and 40%) and the substitution rate of recycled fine aggregates (RFA) (0%, 10%, 20%, 30%) on the axial compressive strength, elastic modulus, and stress–strain behavior of the concrete. The study investigated how the addition of LS and RFA influences the mechanical properties and the stress–strain curve of the concrete under uniaxial compression.	20%	(1) Compressive strength: At 20% LS content, the concrete achieved the highest compressive strength. (2) The incorporation of LS at 20% significantly improved the elastic modulus and peak strain of the concrete. (3) Microstructure: The addition of LS helped refine the cement pores and densify the interfacial transition zone (ITZ), improving the overall durability and performance of the concrete.
[47]	Normal concrete—30 MPa	0%, 10%, 20%, and 30%	Examined the mechanical properties of LSC after exposure to high temperatures, including compressive, axial compressive, and flexural strengths	20%	(1) 20% LS increased strength up to 8.16% after high-temperature exposure. (2) Mass loss was minimal at 20% LS after high-temperature treatment. (3) 20% LS provided a 13.46% increase in flexural strength.
[30]	Autoclaved aerated concrete (AAC)	50% to 75%	Investigated the physical properties and hydration characteristics of autoclaved aerated concrete (AAC) using lithium slag as a replacement for traditional siliceous materials like quartz sand. The study evaluated the compressive strength, bulk density, thermal conductivity, water absorption, and microscopic properties of the resulting AAC.	50–75% lithium slag substitution by mass led to better performance in terms of compressive strength, thermal conductivity, and water absorption.	(1) 50–75% substitution of lithium slag improved compressive strength, with up to 108% of the strength of control specimens. (2) Lithium slag improved the reactivity of the mixture, resulting in better hydration products. (3) Optimal lithium slag content reduced the bulk density and thermal conductivity of the AAC. (4) Lithium slag inclusion reduced water absorption and improved pore structure.
[28]	Normal concrete—45 MPa	20%, 40%, and 60%	Workability, compressive strength, volume of permeable voids (VPV), water penetration depth, sorptivity, and porosity of concrete.	40%	(1) Compressive strength: Concrete containing 40% LS showed the highest compressive strength at 180 days, with 107% strength development compared to the early curing stage (7 days). (2) Porosity and VPV: The porosity and volume of permeable voids were significantly reduced with 40% LS, leading to improved durability and water resistance. VPV decreased by 31.3% at 180 days compared to early-age measurements. (3) Water penetration depth: The water penetration depth was reduced by 36% at 180 days for the 40% LS concrete mix. (4) Sorptivity: The sorptivity coefficient decreased by 75.7% for the 40% LS concrete at 180 days.
[27]	Normal concrete—40 MPa	20%, 40%, and 60%	The study measured compressive strength, split tensile strength, flexural strength, and elastic modulus of the concrete at 7, 28, and 90 days. The microstructure was analyzed using SEM and EDS.	40%	(1) The compressive strength of concrete with 40% LS replacement at 90 days reached 58.6 MPa, which was significantly higher than the 44.8 MPa of the control concrete. (2) 40% LS concrete also showed improvements in split tensile strength (4.11 MPa), elastic modulus (43.92 GPa), and flexural strength (5.4 MPa) at 90 days. (3) Microstructure analysis revealed that the incorporation of lithium slag improved the interfacial transition zone (ITZ), with reduced microcracks and voids, contributing to better mechanical performance during both early and later stages.
[6]	Ultra-high-performance concrete (UHPC)	10%, 20%, 30% and 40%	Investigated the hydration process, microstructure development, and environmental benefits of lithium slag (LS) in UHPC. Various tests, including isothermal calorimetry, X-ray diffraction (XRD), thermogravimetric analysis (TG), scanning electron microscopy (SEM), and mercury intrusion porosimetry (MIP), were conducted.	30%	(1) 30% LS replacement provided an optimal balance between performance and sustainability. (2) 20% LS resulted in the highest 28-day compressive strength of 134.48 MPa. (3) LS acted as a filler, promoted nucleation, and enhanced internal curing. (4) Reduced carbon emissions and improved hydration properties.

## Data Availability

No new data were created or analyzed in this study. Data sharing is not applicable to this article.

## Abbreviations

The following abbreviations are used in this manuscript:

LS	Lithium slag
OPC	Ordinary Portland cement
SCM(s)	Supplementary cementitious material(s)
GGBFS	Ground granulated blast furnace slag
AAC	Autoclaved aerated concrete
UHPC	Ultra-high-performance concrete
HPC	High-performance concrete
RCC	Roller-compacted concrete
SCC	Self-compacting concrete
WRPC	White reactive powder concrete
C–S–H	Calcium silicate hydrate
C–A–S–H	Calcium aluminosilicate hydrate
VPV	Volume of permeable voids
WRC	Water retention capacity
SP(s)	Superplasticizer(s)
UCS	Unconfined compressive strength
RDME	Relative dynamic modulus of elasticity
TGA	Thermogravimetric analysis
SEM	Scanning electron microscopy
EDS	Energy-dispersive X-ray spectroscopy
LCA	Life-cycle assessment

## References

[1] Luo Q., Wen Y.F., Huang S.W., Peng W.L., Li J.Y., Zhou Y.X. (2017). Effects of Lithium Slag from Lepidolite on Portland Cement Concrete: Qi Luo Yufeng Wen, Shaowen Huang, Weiliang Peng, Jinyang Li & Yuxuan Zhou. Civil, Architecture and Environmental Engineering.

[2] He Z., Du S., Chen D. (2018). Microstructure of Ultra High Performance Concrete Containing Lithium Slag. J. Hazard Mater..

[3] Griffin J. (2019). Lithium-Ion Batteries. Electr. Contract..

[4] Zhai M., Zhao J., Wang D., Wang Y., Wang Q. (2021). Hydration Properties and Kinetic Characteristics of Blended Cement Containing Lithium Slag Powder. J. Build. Eng..

[5] Tabelin C.B., Dallas J., Casanova S., Pelech T., Bournival G., Saydam S., Canbulat I. (2021). Towards a Low-Carbon Society: A Review of Lithium Resource Availability, Challenges and Innovations in Mining, Extraction and Recycling, and Future Perspectives. Miner. Eng..

[6] Yang B., Zhang Y., Zhang W., Sun H., Wang Q., Han D. (2024). Recycling Lithium Slag into Eco-Friendly Ultra-High Performance Concrete: Hydration Process, Microstructure Development, and Environmental Benefits. J. Build. Eng..

[7] Rahman S.M.A., Dodd A., Khair S., Shaikh F.U.A., Sarker P.K., Hosan A. (2023). Assessment of Lithium Slag as a Supplementary Cementitious Material: Pozzolanic Activity and Microstructure Development. Cem. Concr. Compos..

[8] Liu Z., Wang J., Li L., Wang D. (2019). Characteristics of Alkali-Activated Lithium Slag at Early Reaction Age. J. Mater. Civ. Eng..

[9] Adediran A., Rajczakowska M., Steelandt A., Novakova I., Cwirzen A., Perumal P. (2025). Conventional and Potential Alternative Non-Conventional Raw Materials Available in Nordic Countries for Low-Carbon Concrete: A Review. J. Build. Eng..

[10] Gao W., Jian S., Li X., Tan H., Li B., Lv Y., Huang J. (2022). The Use of Contaminated Soil and Lithium Slag for the Production of Sustainable Lightweight Aggregate. J. Clean Prod..

[11] Zetola V., Keith B.F., Lam E.J., Montofré Í.L., Rojas R.J., Marín J., Becerra M. (2024). From Mine Waste to Construction Materials: A Bibliometric Analysis of Mining Waste Recovery and Tailing Utilization in Construction. Sustainability.

[12] El Machi A., El Berdai Y., Mabroum S., Safhi A.E.M., Taha Y., Benzaazoua M., Hakkou R. (2024). Recycling of Mine Wastes in the Concrete Industry: A Review. Buildings.

[13] Zabielska-Adamska K. (2025). Industrial By-Products and Waste Materials in Geotechnical Engineering Applications. Emerging Trends in Sustainable Geotechnics: Keynote Volume of EGRWSE 2024.

[14] Villagran-Zaccardi Y., Carreño F., Granheim L., Espín de Gea A., Smith Minke U., Butera S., López-Martínez E., Peys A. (2024). Valorisation of Aggregate-Washing Sludges in Innovative Applications in Construction. Materials.

[15] Saluja S., Gaur A., Somani P., Abbas S. (2025). Use of Stabilized Waste Soil in the Construction of Sustainable Concrete. Recent Developments and Innovations in the Sustainable Production of Concrete.

[16] Zhu J.F., Wang Z.Q., Tao Y.L., Ju L.Y., Yang H. (2024). Macro–Micro Investigation on Stabilization Sludge as Subgrade Filler by the Ternary Blending of Steel Slag and Fly Ash and Calcium Carbide Residue. J. Clean Prod..

[17] Li K., Wang X., Wang X., Tu S., Song Y., Shi T., Wang L., Zhou H. (2025). A Comprehensive Benefit Evaluation of Recycled Carbon Fiber Reinforced Cement Mortar Based on Combined Weighting. Constr. Build. Mater..

[18] Chen Y., Zhang L., Xu L., Zhou S., Luo B., Ding K. (2025). In-Situ Investigation on Dynamic Response of Highway Transition Section with Foamed Concrete. Earthq. Eng. Eng. Vib..

[19] Luo B., Su Y., Ding X., Chen Y., Liu C. (2025). Modulation of Initial CaO/Al_2_O_3_ and SiO_2_/Al_2_O_3_ Ratios on the Properties of Slag/Fly Ash-Based Geopolymer Stabilized Clay: Synergistic Effects and Stabilization Mechanism. Mater. Today Commun..

[20] Guo C., Wang R. (2023). Utilizing Lithium Slag to Improve the Physical-Chemical Properties of Alkali-Activated Metakaolin-Slag Pastes: Cost and Energy Analysis. Constr. Build. Mater..

[21] Zhang L., Pan Y., Xu K., Bi L., Chen M., Han B. (2022). Corrosion Behavior of Concrete Fabricated with Lithium Slag as Corrosion Inhibitor under Simulated Acid Rain Corrosion Action. J. Clean Prod..

[22] Begentayev M.M., Nurpeisova M.B., Fedotenko V.S. (2020). The use of mining and metallurgy waste in manufacture of building materials. Tech. Sci..

[23] Bueno-Gómez T., López-Bernier Y., Caycedo-García M.S., Ardila-Rey J.D., Rodríguez-Caicedo J.P., Joya-Cárdenas D.R. (2025). Valorization of Gold Mining Tailings Sludge from Vetas, Colombia as Partial Cement Replacement in Concrete Mixes. Buildings.

[24] Wen H. (2013). Property Research of Green Concrete Mixed with Lithium Slag and Limestone Flour. Adv. Mat. Res..

[25] Wu F.F., Shi K.B., Dong S.K. (2014). Influence of Concrete with Lithium-Slag and Steel Slag by Early Curing Conditions. Key Eng. Mater..

[26] Gu T., Zhang G., Wang Z., Liu L., Zhang L., Wang W., Huang Y., Dan Y., Zhao P., He Y. (2024). The Formation, Characteristics, and Resource Utilization of Lithium Slag. Constr. Build. Mater..

[27] Rahman S.M.A., Shaikh F.U.A., Sarker P.K. (2024). Fresh, Mechanical, and Microstructural Properties of Lithium Slag Concretes. Cem. Concr. Compos..

[28] Amin M.T.E., Sarker P.K., Shaikh F.U.A. (2024). Transport Properties of Concrete Containing Lithium Slag. Constr. Build. Mater..

[29] Chen X.-B., Liang J.-F., Li W. (2024). Compression Stress-Strain Curve of Lithium Slag Recycled Fine Aggregate Concrete. PLoS ONE.

[30] Wang S., Gu X., Liu J., Zhu Z., Wang H., Ge X., Hu Z., Xu X., Nehdi M.L. (2024). Assessment of Lithium Slag as a Supplementary Siliceous Material in Autoclaved Aerated Concrete: Physical Properties and Hydration Characteristics. Constr. Build. Mater..

[31] Li B., Cao R., You N., Chen C., Zhang Y. (2019). Products and Properties of Steam Cured Cement Mortar Containing Lithium Slag under Partial Immersion in Sulfate Solution. Constr. Build. Mater..

[32] Li J., Huang S. (2020). Recycling of Lithium Slag as a Green Admixture for White Reactive Powder Concrete. J. Mater. Cycles Waste Manag..

[33] Luo Q., Huang S., Zhou Y., Li J., Peng W., Wen Y. (2017). Influence of Lithium Slag from Lepidolite on the Durability of Concrete. Proceedings of the IOP Conference Series: Earth and Environmental Science.

[34] He Y., Zhang Q., Chen Q., Bian J., Qi C., Kang Q., Feng Y. (2021). Mechanical and Environmental Characteristics of Cemented Paste Backfill Containing Lithium Slag-Blended Binder. Constr. Build. Mater..

[35] Foghi E.J., Vo T., Rezania M., Nezhad M.M., Ferrara L. (2024). Early Age Hydration Behaviour of Foam Concrete Containing a Coal Mining Waste: Novel Experimental Procedures and Effects of Capillary Pressure. Constr. Build. Mater..

[36] Silva Y.F., Burbano-Garcia C., Araya-Letelier G., Izquierdo S. (2025). Sulfate Attack Performance of Concrete Mixtures with Use of Copper Slag as Supplementary Cementitious Material. Case Stud. Constr. Mater..

[37] Li T., Tang X., Xia J. (2024). Functional Characteristics of Sustainable Pervious Cement Concrete Pavement Modified by Silica Fume and Travertine Waste. Ceram.–Silikáty.

[38] Gou H., Rupasinghe M., Sofi M., Sharma R., Ranzi G., Mendis P., Zhang Z. (2023). A Review on Cementitious and Geopolymer Composites with Lithium Slag Incorporation. Materials.

[39] de Souza E.A., de Kássia Rodrigues e Silva R., Borges P.H.R. (2024). Overburden Materials from the Iron Mining as Raw Materials for AAM: Preliminary Assessment of Mixes for 3D Concrete Printing. Proceedings of the FIB International Conference on Concrete Sustainability.

[40] Mim N.J., Shaikh F.U.A., Sarker P.K. (2025). Sustainable 3D Printed Concrete Incorporating Alternative Fine Aggregates: A Review. Case Stud. Constr. Mater..

[41] Rahman S.M.A., Mahmood A.H., Shaikh F.U.A., Sarker P.K. (2023). Fresh State and Hydration Properties of High-Volume Lithium Slag Cement Composites. Mater. Struct..

[42] Sousa J.T.F.d., Anjos M.A.S.d., Neto J.A.d.S., Farias E.C.d., Branco F.G., Maia Pederneiras C. (2025). Self-Compacting Concrete with Artificial Lightweight Aggregates from Sugarcane Ash and Calcined Scheelite Mining Waste. Appl. Sci..

[43] Cuenca E., Del Galdo M., Aboutaybi O., Ramos V., Nash W., Rollinson G.K., Andersen J., Crane R., Ghorbel E., Ferrara L. (2024). Mechanical Characterization of Cement Mortars and Concrete with Recycled Aggregates from Coal Mining Wastes Geomaterials (CMWGs). Constr. Build. Mater..

[44] Wu F.F., Shi K.B., Dong S.K. (2014). Properties and Microstructure of HPC with Lithium-Slag and Fly Ash. Key Eng. Mater..

[45] Vigneshwari A., Jayaprakash J. (2024). A Review on 3D Printable Cementitious Material Containing Copper and Iron Ore Tailings: Material Characterization, Activation Methods, Engineering Properties, Durability, and Microstructure Behavior. Innov. Infrastruct. Solut..

[46] Yu H., Zahidi I., Chow M.F., Liang D., Madsen D.Ø. (2024). Reimagining Resources Policy: Synergizing Mining Waste Utilization for Sustainable Construction Practices. J. Clean Prod..

[47] Liang J., Zou W., Tian Y., Wang C., Li W. (2024). Effect of High Temperature on Mechanical Properties of Lithium Slag Concrete. Sci. Rep..

[48] Zhai J., Chen P., Long J., Fan C., Chen Z., Sun W. (2024). Recent Advances on Beneficial Management of Lithium Refinery Residue in China. Miner. Eng..

[49] Javed U., Shaikh F.U.A., Sarker P.K. (2024). Corrosive Effect of HCl and H2SO4 Exposure on the Strength and Microstructure of Lithium Slag Geopolymer Mortars. Constr. Build. Mater..

[50] He Y., Kang Q., Lan M., Peng H. (2023). Mechanism and Assessment of the Pozzolanic Activity of Melting-Quenching Lithium Slag Modified with MgO. Constr. Build. Mater..

[51] Zhang Y., Yang B., Gu X., Han D., Wang Q. (2023). Improving the Performance of Ultra-High Performance Concrete Containing Lithium Slag by Incorporating Limestone Powder. J. Build. Eng..

[52] Khair S., Rahman S.M.A., Shaikh F.U.A., Sarker P.K. (2024). Evaluating Lithium Slag for Geopolymer Concrete: A Review of Its Properties and Sustainable Construction Applications. Case Stud. Constr. Mater..

[53] He Y., You C., Jiang M., Liu S., Shen J., Hooton R.D. (2023). Rheological Performance and Hydration Kinetics of Lithium Slag-Cement Binder in the Function of Sodium Sulfate. J. Therm. Anal. Calorim..

[54] Zhang Y., Zhang L., Wang Q., Han D., Li Z. (2023). Iron Ore Tailings, Phosphate Slags, and Lithium Slags as Ternary Supplementary Cementitious Materials for Concrete: Study on Compression Strength and Microstructure. Mater. Today Commun..

[55] Luo Q., Liu Y., Dong B., Ren J., He Y., Wu K., Wang Y. (2023). Lithium Slag-Based Geopolymer Synthesized with Hybrid Solid Activators. Constr Build Mater.

[56] Zhou M., Yan J., Fan J., Xu Y., Lu Y., Duan P., Zhu Y., Zhang Z., Lu Z. (2023). Insight to Workability, Compressive Strength and Microstructure of Lithium Slag-Steel Slag Based Cement under Standard Condition. J. Build. Eng..

[57] Tan H., Li M., He X., Su Y., Zhang J., Pan H., Yang J., Wang Y. (2020). Preparation for Micro-Lithium Slag via Wet Grinding and Its Application as Accelerator in Portland Cement. J. Clean Prod..

[58] Qin Y., Chen J., Li Z., Zhang Y. (2019). The Mechanical Properties of Recycled Coarse Aggregate Concrete with Lithium Slag. Adv. Mater. Sci. Eng..

[59] Chen L., Yao J., Zhang G. (2018). Flexural Properties of Lithium Slag Concrete Beams Subjected to Loading and Thermal-Cold Cycles. KSCE J. Civ. Eng..

[60] Tan H., Zhang X., He X., Guo Y., Deng X., Su Y., Yang J., Wang Y. (2018). Utilization of Lithium Slag by Wet-Grinding Process to Improve the Early Strength of Sulphoaluminate Cement Paste. J. Clean Prod..

[61] He Z., Li L., Du S. (2017). Mechanical Properties, Drying Shrinkage, and Creep of Concrete Containing Lithium Slag. Constr. Build. Mater..

[62] Qin Y., Chen J., Liu K., Lu Y. (2021). Durability Properties of Recycled Concrete with Lithium Slag under Freeze-Thaw Cycles. Mater. Technol..

[63] Shi K.B., Zhang S. (2011). De Ring Method Test on the Early-Age Anti-Cracking Capability of High-Performance Lithium Slag Concrete. Appl. Mech. Mater..

[64] He Z., Chang J., Du S., Liang C., Liu B. (2020). Hydration and Microstructure of Concrete Containing High Volume Lithium Slag. Mater. Express.

[65] Rahman S.A., Shaikh F.U.A., Sarker P.K. (2022). A Comprehensive Review of Properties of Concrete Containing Lithium Refinery Residue as Partial Replacement of Cement. Constr. Build. Mater..

[66] Ding X.H., Luo B., Zhou H.T., Chen Y.H. (2025). Generalized Solutions for Advection–Dispersion Transport Equations Subject to Time- and Space-Dependent Internal and Boundary Sources. Comput. Geotech..

